# Identification of Relevant Protein Interactions with Partial Knowledge: A Complex Network and Deep Learning Approach

**DOI:** 10.3390/biology12010140

**Published:** 2023-01-16

**Authors:** Pilar Ortiz-Vilchis, Jazmin-Susana De-la-Cruz-García, Aldo Ramirez-Arellano

**Affiliations:** 1 Sección de Estudios de Posgrado e Investigación, Escuela Superior de Medicina, Instituto Politécnico Nacional, Mexico City 11340, Mexico; 2 Sección de Estudios de Posgrado e Investigación, Unidad Profesional Interdisciplinaria de Ingeniería y Ciencias Sociales y Administrativas, Instituto Politécnico Nacional, Mexico City 08400, Mexico

**Keywords:** scale-free, fractals, complex networks, protein–protein interactions networks, bidirectional LSTM

## Abstract

**Simple Summary:**

Protein–protein interactions (PPIs) are the basis for understanding cellular events in biological systems. Experimental biochemical, molecular, and genetic methods have been used to identify protein–protein associations. However, they are time-consuming and expensive. Machine learning techniques have been used to characterize PPIs, optimizing time and resources. This study aimed to generate a relevant protein sequence with partial knowledge of interactions by conducting a scale-free and fractal analysis. The outcome of these analyses is then used to fine-tune the fractal method for the vital protein extraction of PPI networks. The results show that several PPI networks are self-similar or fractal, but not both of them. The generated protein sequences by the deep learning network contains an important number of proteins of the original sequence. Moreover, most of the PPIs of generated sequences appear in the original set. This information can help researchers guide experimental design and find key points for new therapeutics.

**Abstract:**

Protein–protein interactions (PPIs) are the basis for understanding most cellular events in biological systems. Several experimental methods, e.g., biochemical, molecular, and genetic methods, have been used to identify protein–protein associations. However, some of them, such as mass spectrometry, are time-consuming and expensive. Machine learning (ML) techniques have been widely used to characterize PPIs, increasing the number of proteins analyzed simultaneously and optimizing time and resources for identifying and predicting protein–protein functional linkages. Previous ML approaches have focused on well-known networks or specific targets but not on identifying relevant proteins with partial or null knowledge of the interaction networks. The proposed approach aims to generate a relevant protein sequence based on bidirectional Long-Short Term Memory (LSTM) with partial knowledge of interactions. The general framework comprises conducting a scale-free and fractal complex network analysis. The outcome of these analyses is then used to fine-tune the fractal method for the vital protein extraction of PPI networks. The results show that several PPI networks are self-similar or fractal, but that both features cannot coexist. The generated protein sequences (by the bidirectional LSTM) also contain an average of 39.5% of proteins in the original sequence. The average length of the generated sequences was 17% of the original one. Finally, 95% of the generated sequences were true.

## 1. Introduction

Protein–protein interactions (PPIs) are the basis for understanding most cellular events in biological systems. Several methods have been used to identify protein–protein associations, to study and understand a cell’s physiological activities, such as signal transduction, transcriptional regulation, and metabolic and regulatory pathways, and even to investigate therapeutic targets. Experimental methods, such as biochemical methods in cell cultures [1] and living organisms [2], have been used to determine direct interactions in order to evaluate binding affinities in real time [3], examine pathogens’ virulence [4], quantify and visualize PPIs in cells and tissues [5], and understand the nature of PPIs during biogenesis reactions [6]. Moreover, molecular methodologies have been included to detect specific PPIs and develop antifungals that disrupt virulence [7], characterize and screen protein–protein complexes in a model antibody-antigen system [8], map and quantify effector–host PPIs during an infection [9], and detect and characterize PPIs in vivo and in vitro assays [10]. Finally, genetic approaches have been used to identify phase mutations (G2/M or G1/S) regulated by protein–protein interactions on eukaryotic cells [11], understand the cellular construction of nanostructures through protein–protein interactions [12], identify the physical interactions and screen mutation function of some enzymes in a yeast network [13], and detect genetic interactions as potential anticancer therapeutic targets [14]. However, most of them are time-consuming and expensive.

Additionally, in silico approaches allow for modeling molecular interactions [15], testing conformational changes of protein–protein docking and protein–DNA docking [16], detecting enzyme activity [17], structurally characterizing two different molecules [18], and even designing new therapeutics. In the same way, datasets are used to identify functional interactions and detect likely PPIs [19], infer functionally similar genes, and understand the pathogenesis of the disease [20]. Nevertheless, experimental and computational methods are individually designed and are carried out for specific interactions.

Machine learning (ML) techniques have been widely used for characterizing sequences of PPIs [21,22], considering the amino acid residue as the interaction site [23] and transforming biological sequences into numerical representations [24], thus increasing the number of proteins analyzed simultaneously and optimizing time and resources. In most cases, different ML approaches have been used in computing PPI networks, using well-known physiochemical properties and evolutionary profiles. However, to the best of our knowledge, all investigations have focused on well-known networks or specific targets, not on identifying relevant proteins with partial or null knowledge of the interaction network. This work aims to generate a relevant protein sequence based on bidirectional Long-Short Term Memory (LSTM) without knowledge of their specific interactions. The proposed approach has roots in the complex network analysis pursuing two purposes: to give evidence that several PPI networks are fractal but not scale-free and to extract the relevant proteins based on fractality. The relevant protein sequences (extracted from known PPI networks in which target proteins partake) are the cornerstone to building a bidirectional LSTM network; the LSTM will then generate a sequence based on target proteins.

Related work and preliminaries that underpin this research will be introduced below, followed by a presentation of the methodology and the results. The discussion and conclusion will be given afterwards.

## 2. Related Work

ML techniques have been used on molecular and cellular levels to model, identify, and predict binding interactions. The support vector machine algorithm has been used to predict interactions of a pair of proteins [25,26]. Deep-learning neural networks have been employed to design novel peptides [27]. On the other hand, based on physical and semantic information about amino acids, the support vector machine classifies the sequences (of a fixed length *n* and a set of 20 amino acids) [28] as positive (they exist) or negative. Similarly, for predicting host–pathogen PPIs, an LSTM was developed to identify the positive sequence of amino acids [29]. The approaches in [28,29] have a high accuracy, of more than 0.98. An LSTM can also identify matches of PPIs from four different species with prediction accuracies of more than 0.92 (rodent: 0.92; bacterium: 0.96; fly: 0.98; nematode: 0.99) [30]. In the same way, PPIs of primary amino acid sequences across species were identified in [31]. The deep neural network provides the probability that a pair of proteins interact, and these candidate interactions are compared with those that occur to evaluate the performance. The precision ranged from 0.51 to 0.58, and the recall ranged from 0.22 to 0.54, depending on the species.

Furthermore, classical ML algorithms such as naive Bayes and the support vector machine have been employed to differentiate expressed genes [32,33] and validate gene biomarkers [34]. These approaches used relevant nodes from PPI networks that usually are extracted based on centrality measures such as node degree, closeness, and betweenness. Moreover, ML tools have helped to classify diseases [35] and prognostic mutations [36] and detect molecular diseases [37] based on PPI, as well as to identify infectious diseases and the PPIs between humans and viruses [38,39,40]. Furthermore, clustering methods on PPI networks have been employed to construct hierarchy trees and detect functional modules [41].

Complex network analysis, such as the fractal dimension of PPI networks, has been employed to detect the sets of PPIs that form subnetworks. In this approach, the fractal dimension is the clustering metric that considers the number of nodes and edges in the boxes computed by the sandbox algorithm [42]. Furthermore, the fuzzy fractal dimension of PPI networks has been used to identify the essential proteins in PPI networks [43]. The crucial scale-free property of the dementia and hereditary Parkinson’s PPI networks emerges when the vital proteins are deleted from them, revealing their importance not only in the biological process but also in the network’s topology [44].

The previous work shows that the ML approaches infer potential interactions, validate previous results, and analyze PPI networks. Deep learning techniques, such as LSTM, and classical machine learning, such as support vector machine algorithms, have shown that they can classify sequences and discern between positive and negative PPIs. Nevertheless, they cannot create new long sequences, as is the purpose of this work. The new unknown PPI sequences obtained by computational methods could help biologists to guide investigations and reduce research time, experiments, and laboratory consumables, leading to the development, design, and discovery of effective drugs acting on these new interactions.

## 3. Preliminaries

### 3.1. The Scale-Free Property of Protein–Protein Networks

The topology of PPI networks, like complex networks, is influenced by preferential connection, attraction, and repulsion between hub nodes, directionality, and the number of connections [45]. Additionally, complex networks may have small-world and scale-free properties that influence their resilience. The resilience of networks has received relevant attention in recent years [46,47,48]. Scale-free networks are known to be resilient to random attacks but not directed ones, especially to nodes with greater importance. An example of directed attacks is selecting the highest-degree nodes and deleting them.

A network has the property of being scale-free if the degree of the nodes follows a power law distribution:

(1) $$P\left(k\right)=k^{-\alpha },$$

where α > 1 (scale exponent), $k>=k_{min}>=1$.

A power law with exponential cutoff is also a scale-free model, defined as

(2) $$P\left(k\right)=k^{-\alpha }e^{-\lambda k},$$

On the other hand, the networks where the probability distribution of the degree follows an exponential one do not possess a scale-free property.

(3) $$P\left(k\right)=e^{-\lambda k},$$

where λ is the decay exponent.

Furthermore, Weibull and log-normal generalize the exponential distribution defined by Equations (4) and (5), respectively.

(4) $$P\left(k\right)=e^{-{\left(\frac{k}{\lambda }\right)}^{\alpha }},$$

where α is the shape parameter, and λ is the scale parameter.

(5) $$P\left(k\right)=\frac{1}{k}e^{-\frac{{\left(\log k-\mu \right)}^{2}}{2\sigma^{2}}},$$

where μ is the mean, and σ is the standard deviation.

### 3.2. The Fractal Dimension of Protein–Protein Networks

From a geometric perspective, a fractal is an object (for example, a compact set) that is similar to parts of itself [49] with a non-integer Hausdorff dimension that is strictly greater than the topological dimension (it is always an integer) [50]. The box-counting dimension is more appropriate than the Hausdorff dimension to measure the roughness of an object [51]. Based on these ideas, the box-counting dimension ($d_{b}$) for a complex network was introduced in [52,53], and when it follows a power law as

(6) $$N_{b}\left(l\right)∼\beta l^{-d_{b}},$$

the network is said to be a fractal network. $N_{b}\left(l\right)$ is the minimum number of boxes of size *l* needed to cover the network. The box-counting dimension quantifies the pattern’s complexity as a ratio of the change in detail to the change in scale. If the number of boxes follows an exponential function, it is not a fractal network.

(7) $$N_{b}\left(l\right)∼\beta e^{-d_{b}l}.$$

Two models for complex networks with an extra parameter have been proposed [54,55], known as the delayed fractal,

(8) $$N_{b}\left(l\right)∼\beta \frac{\tau +1}{\tau +l^{d_{b}}},$$

and the delayed exponential,

(9) $$N_{b}\left(l\right)∼\beta \frac{\tau +1}{\tau +e^{d_{b}l}},$$

where $d_{b}$ is the box count dimension, β is the scale factor, *l* is the diameter of the boxes to cover the network, and Nb(l) is the number of boxes for Equations (6)–(9). τ >= 0 is known as the delay parameter in Equations (8) and (9).

### 3.3. Extraction of the Relevant Proteins of the Interaction Network

PPI networks are complex [56,57,58] and could have a fractal topology [55]. The fractality is the key to extracting relevant nodes of a network in order to destroy it by identifying network boxes [46]. In a fractal network, the boxes contain a hub (a node where several nodes are connected), and those boxes are usually connected to others by the hub (assortativity). The most relevant nodes, if the network is fragmented, can be identified by eliminating the nodes with the highest betweenness within a box. These nodes are considered relevant. Deleting high-ranked betweenness nodes could make others disconnected; these “satellite” nodes are not regarded as relevant [46]. The fractal methodology performs better than the degree, betweenness, and PageRank methods on fractal and non-fractal networks; for more details; see [46].

**Figure 1:**
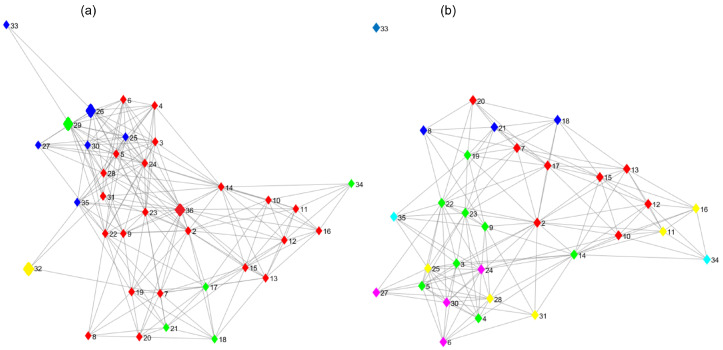
Steps of extracting relevant nodes by the fractal method. The nodes are distributed in four boxes, each color representing a box. (**a**) First extraction step. (**b**) Second extraction step.

Figure 1 is an example of the nodes distributed in four boxes (each color representing a box). The first step in fragmenting the network is to identify and delete the node with the highest betweenness for each box (Node 36, 29, 26, and 32, ordered from the highest to lowest betweenness value; see Figure 1a); consequently, Node 33 turns out to be a satellite node. In the next step, Node 2, 14, 24, 18, and 35 are identified as new relevant nodes. These steps are repeated until there are no connected nodes (Figure 1b).

Extracting vital nodes can identify the relevant proteins of the interaction network. Once an ordered list (from the most relevant to the least) of proteins is obtained, the subnetwork formed by those proteins and their respective arcs contains fewer nodes and arcs than the original one. On the other hand, selecting the nodes with the most connection is an effective method of destruction when the networks are scale-free [59] since these nodes maintain connectivity [60].

## 4. Method

### 4.1. PPI Network Collection

Cytoscape 3.9.1 [61] with the plugin stringApp was used to visualize and retrieve networks from the STRING database [62]. The query was performed on the DISEASES database [61], and the result was exported as a network. Several search terms and suffixes, such as “aortic”, “astro”, “ataxia”, “biotin”, “bull”, “cal”,“cardiac”, “iso”, “tumor”, “type”, “valv”, “veno”, “viral”, and “vitelli” (Table S1 shows all terms and suffixes), were used one by one in different queries. Using two or more terms in the same query produces no results. All PPIs were considered to build the networks, regardless of whether they were experimental or not. A total of 476 human PPI networks were exported from the DISEASES database, and two more were exported from the BioGRID database (https://thebiogrid.org/, last accessed on 30 December 2022) to compare our results using a different source. The networks were retrieved by expanding the number of nodes to the maximum allowed by the Cytoscape stringApp in each query. Finally, networks with more than 101 nodes were selected. The average and standard deviation of the number of nodes were 658.36 and 648.99, respectively; for further detail on the number of nodes of each network; see Table S3. The networks from BioGRID are the last two rows of Tables S2 and S3. Before being analyzed by the scale-free property and fractality, the largest component of each network was selected. An organ is a group of tissues that perform a specific function, and a system is a group of organs that work collectively to accomplish more than one function. Table 1 shows the number of networks grouped manually by the functions of human organs to match networks with proteins in common. The PPI networks could belong to more than one class because some share functions in more than one human organ.

### 4.2. Analysis of the Scale-Free Property and Fractality

The node degree distribution of each PPI network was fitted to the power law model (1), the power law model with a cutoff (2), the exponential model (3), the Weibull model (4), and the log-normal model (5). The best model was selected based on the Akaike Information Criterion (AIC) [63], according to ΔAIC, computed as follows. First, the AIC of *i*th model $AIC_{i}$ was obtained; in our case, *i* stands for the power law model, the power law model with a cutoff, the exponential model, the Weibull model, and the log-normal model. The $\Delta AIC_{i}$ was computed, selecting the minimum AIC over all models tested $AIC_{min}$ and subtracted to each $AIC_{i}$. The model’s ΔAIC with the minimum AIC was 0; thus, this model could be considered the first candidate. Following the rule of thumb [64,65], the first candidate model was different from the others with sufficient statistical evidence (and must be selected as the best) if the ΔAIC is greater than 2. However, the models cannot be differentiated. The AIC selection differs from the likelihood–ratio test employed in [66] since AIC deals with the tradeoff between the goodness of fit and the model’s simplicity. The fit of each model was computed in Matlab R2022a using the fitdist function, except for (1), which was computed using the approach introduced in [67], which searches for $K_{min}$ (described in Equations (1)), which minimizes the distance between the observed data and the power law model. This method was implemented in [66] to conduct an extensive study on real networks. The scale-free analysis provides empirical evidence allowing for the use of the fractal method to obtain the sequence of proteins—instead of deleting the nodes with the most connections (maximum degree-based attack). Comparison with the results of the fractal analysis provides evidence of whether scale-free and fractal properties coexist and can provide evidence that scale-free networks are rare [66,68].

Fractal analysis was carried out on 478 PPI networks. The algorithm employed to compute the minimum number of boxes $N_{b}\left(l\right)$ needed to cover the network was introduced in [69]. The code section of the Supplementary Material contains a MATLAB R2022a implementation of this algorithm and a brief example of its use. Once the dispersion of *l* vs. $N_{b}$ was obtained, the fitnlm function was employed to obtain the AIC. The classification based on this analysis allows us to tune the diameter of the boxes *l* used in the fractal method for relevant node extraction [46]. For example, the fractal networks that follow Equations (6) or (8) can be destroyed efficiently by choosing l=d+1, where *d* is the network’s diameter. The best model of Equations (6)–(9) is chosen by the AIC, as explained above. This approach has been employed in other work to classify complex networks as fractal or non-fractal [46,55,70,71,72]. The fractal analysis shows that 20% of the PPI networks are delayed fractals, and about 80% are delayed exponentials. For the former, l=d+1 of the fractal method was used, and l=d was used for the latter.

### 4.3. Network Architecture for Regression and Protein Sequence Generation

**Figure 2:**
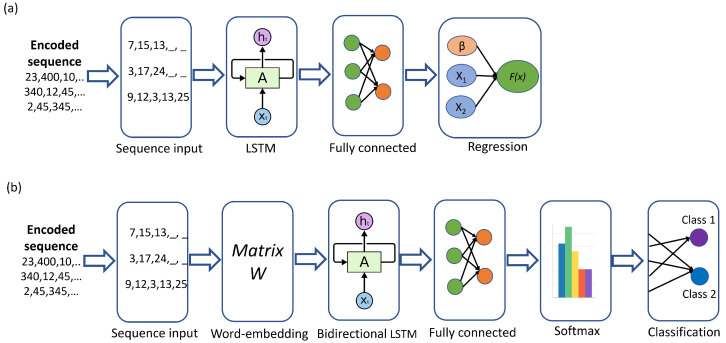
(**a**) LSTM Network architecture for AURC regression. (**b**) Biderectional LSTM Network architecture for generating protein sequences.

The techniques of natural language processing, such as word2vec [73], used to obtain the distributed representation of words have been used in computational biology [74,75,76]. The architecture of the regression LSTM network of AURC is depicted in Figure 2a. The protein sequences are encoded as integer numbers with the wordEncoding MATLAB function; the sequence layer receives a normalized vector of an integer number of these sequences that are the input of the LSTM layer. The LSTM layer contains 350 hidden units. Its output passes to the fully connected layer that connects all inputs to the outputs with weights and biases. The dropout layer with a probability of 0.2 is between the LSTM and the fully connected layer to avoid over-fitting. The regression layer computes the half-mean-squared-error loss for regression tasks and computes the responses—in our case, the AURC.

The sequences of nodes (proteins) obtained by the fractal and maximum degree-based method were threatened as a sequence of “words”, where each word is an integer number that identifies a protein. Both encoded sequences of proteins were then compared using the regression LSTM to predict the Area Under the Resilience Curve (AURC) [46] to show evidence that the fractal method outperforms the maximum degree-based attack. The fit of the regression LSTM was compared using the Mean Absolute Percentage Error (MAPE), Mean Absolute Error (MAE), Root Mean Square Error (RMSE), and adjusted correlation coefficient ($R^{2}adj$).

The bidirectional LSTM architecture for generating new sequences; see Figure 2b, consists of an input sequence layer that receives the encoded protein sequence with the wordEncoding MATLAB function. The training sequences are those extracted by the fractal method of the networks grouped by functions of human organs; see Table 1. The word-embedding layer (dimension = 100) maps word indices to vectors that feed the bidirectional LSTM layer with 350 hidden units. A fully connected layer follows the bidirectional LSTM. The bidirectional LSTM can employ the information of both sides of the sequence (backwards and forward), instead of only one side, as in LSTM. The bidirectional LSTM outperforms the LSTM when full sequences are processed ([77], p. 107). The softmax layer smoots the outputs of the bidirectional LSTM to warrant that the probabilities that all possible proteins amount to 1. Finally, the classification layer computes the cross-entropy loss for each generated sequence of proteins.

**Figure 3:**
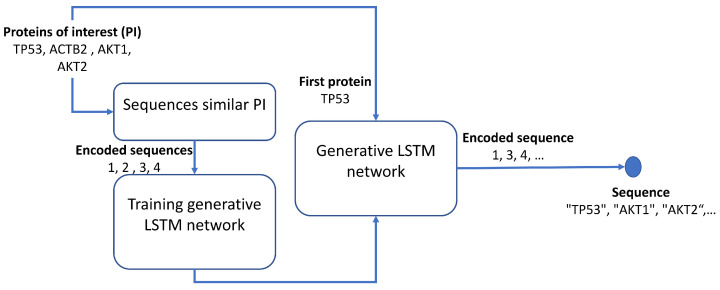
The generative process of protein sequences by bidirectional LSTM.

In the training process, a sequence from those grouped by the functions of human organs (training set) is chosen. The training set is refined by selecting the sequences that contain proteins of the selected sequence (sequences whose Jaccard coefficient is above the threshold of 0.15); see Figure 3. Furthermore, the selected sequence is removed from the training set. For example, let “TP53”, “ACTB2”, “AKT1”, and “AKT2” be the Proteins of Interest (PIs) of the selected sequence. Once the LSTM network is trained, the generative process starts with the first PI, such as “TP53”, and in each step, the bidirectional LSTM network gives a scored set of candidate proteins, from which the highest score protein is identified and added to the new sequence. The protein generated in the previous step is now the seed. These steps are repeated until the number of proteins generated equals the number of proteins in the real sequences. The process can stop before the length of the real sequences is reached if the trained LSTM networks cannot find a new protein. In other words, there is no protein with a probability higher than zero, or the protein is already in the generated sequences. In our brief example, “AKT1” is added in the second step, and “AKT2” is added in the third. This new sequence is an ordered list (relying on the score) of relevant proteins. The training of the LSTM network and generation process is repeated for each sequence in the training set. The regression LSTM and the bidirectional LSTM networks were implemented in MATLAB R2022a. The example code to generate a new protein sequence can be found in the Supplementary Material.

In evaluating the accuracy of the generative process, the Jaccard coefficient and the Levenshtein distance [78] between the generated sequence and the original sequence are computed. The first quantifies how similar the two sequences of proteins are (as a set) but neglects the position of each protein in the sequence. The Levenshtein distance fills this gap. For example, let “TP53”, “AKT2”, “ACTB2” be the original sequence, and let “TP53”, “ACTB2”, “AKT2” be the generated sequences. The Jaccard coefficient between them is 1. However, the proteins are in a different order. The generated sequence can be transformed into the original, changing “AKT2” to “ACTB2” and “ACTB2” to “AKT2”. The Levenshtein distance quantifies these two operations. Hence, the larger the value of the Levenshtein distance, the greater the difference between the two sequences.

## 5. Results

The scale-free analysis shows that only four PPI network nodes’ degrees follow a power law with a cutoff distribution; 161 were exponential, and for 30 PPI networks, there is no sufficient statistical evidence supporting a choice between exponential and log-normal models. Finally, for 281 PPI networks, the node’s degree follows a log-normal distribution; see Table S2. Table 2 summarizes these results, showing that most PPI networks follow a log-normal distribution.

**Figure 4:**
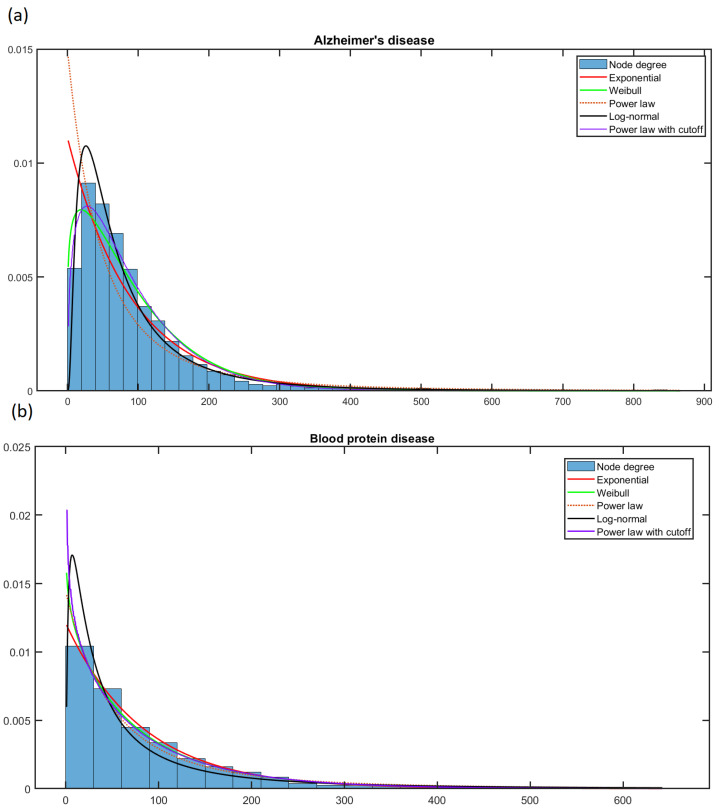
The fit of several models for the node degree probability distribution of (**a**) Alzheimer’s network and (**b**) blood protein disease network.

Figure 4 shows the fit of five models for the node degree of (a) Alzheimer’s and (b) Blood protein (hyperproteinemia and hypoproteinemia) diseases. Moreover, Figure S1 of Supplementary Material shows the fit of several models for the node degree probability distribution of (a) Endocarditis and (b) the Gilles de la Tourette syndrome network. Figure 4 and Figure S1 reveal that selecting the best without the AIC is rather difficult. The scale-free analysis results show that 92.88% of the degree distributions of the PPI networks in this work follow a kind of exponential distribution (exponential and log-normal); thus, they are not scale-free. These results undermine the use of the maximum degree-based attack since it is the preferable method for obtaining relevant nodes if the network has the scale-free property.

The fractal analysis shows that the box-covering of 57.74% of PPI networks best fits the delayed exponential function, that of 20.29% is best for the delayed fractal, and that of only 2.30% is best for the exponential function. The number of networks that cannot be differentiated between exponential or delayed exponential or between exponential or fractal is reported in Table 3. Thus, fractal networks (20.29%) are not as rare as self-similar ones (0.84%). Moreover, the self-similar and fractal analyses suggest that fractality and self-similarity cannot coexist in the PPI networks; see Tables S2 and S3.

**Figure 5:**
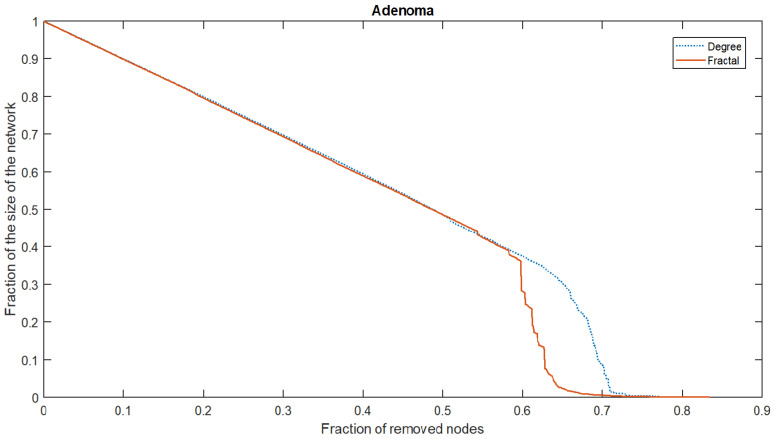
The resilience curve of the Adenoma PPI network was obtained by removing the proteins using fractal and maximum degree-based methods.

The box-covering of the PPI networks is mostly of the exponential type (78.87–57.74% delayed exponential, 2.30% exponential, and 18.83% exponential or delayed exponential), and 20.29% is of the delayed fractal type; meanwhile, only four cannot be classified in one of the previous sets. These results, in conjunction with the self-similar analysis, suggest that the fractal method for obtaining the relevant proteins of the PPI network is the most suitable, since it obtains good results in fractal and non-fractal networks. For more evidence supporting this, the relevant proteins obtained by the maximum degree-based method and their correlation with the resilience of the PPI network (measured by AURC) [46] were compared with that obtained by the fractal method. The regression LSTM network was employed for this purpose. An example of the AURC is shown in Figure 5. The fraction of nodes removed was plotted vs. the fraction of the size of the largest component in the network. Initially, the size is 1, and the fraction of the removed nodes is 0. For a resilient network, the AURC will be approximately 0.5, since the resilience curve will be a straight line with a slope of $-\frac{1}{2}$. On the contrary, an AURC closer to 0 means that the network’s resilience is poor. The AURC of the fractal and maximum degree-based attacks on the same network provide a measure of their effectiveness that can be compared; for example, the method with the lowest AURC is the most effective at destroying the network. The AURC was computed when the relevant nodes were obtained by the fractal and maximum degree-based methods. A *t*-test shows that the fractal method obtained a lower AURC (μ=0.288, σ=0.09) compared with the maximum degree-based method (μ=0.35, σ=0.073), t(475)=48.316 p<0.0001. Hence, the proteins obtained by the fractal method are more suitable for maintaining the cohesion of the PPI network.

The MAPE, MAE, and RMSE of the regression of the protein sequences and the AURC obtained by the fractal method are significantly lower than those of the maximum degree-based method. On the other hand, the $R^{2}adj$ of the fractal method is higher than the $R^{2}adj$ of the maximum degree-based method; see Table 4. The previous results support the finding that the sequences extracted by the fractal method are suitable for maintaining the cohesion of PPI networks. Furthermore, these protein sequences are highly correlated with the PPI network’s resilience.

**Figure 6:**
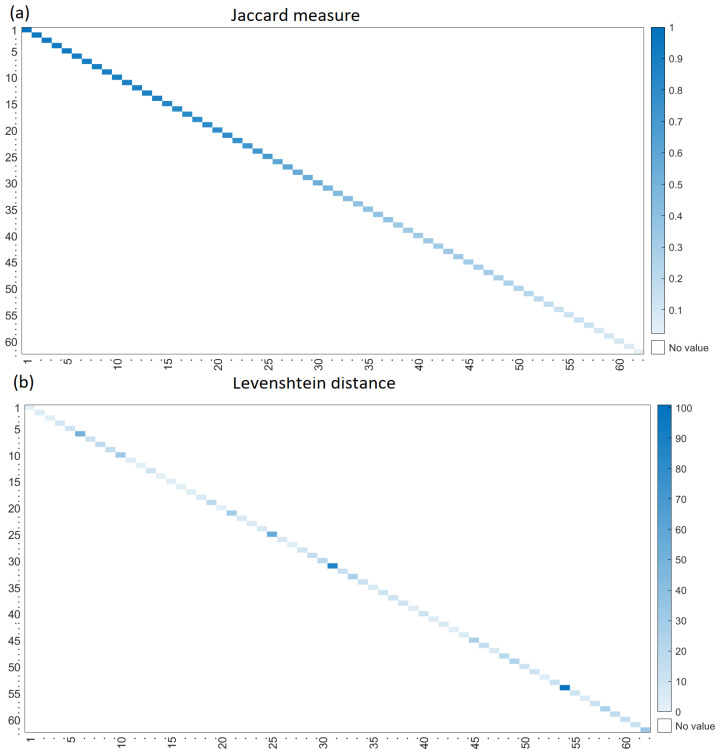
The (**a**) Jaccard measure and (**b**) the Levenshtein distance between the real and generated sequences of Bone.

The bidirectional LSTM network was then trained, as described above. The accuracy of the generation process was tested using the protein sequences grouped in Immune, Metabolism, Motor, Nerve, and Bone functions; see Table 1. The generated and the original sequences were compared in terms of the Jaccard measure and Levenshtein distance. Both sequences were expected to be identical, so the Jaccard value was 1 in this case, and the Levenshtein distance was 0. Since the length of the generated sequences varied from 2 to the length of the original sequence, those with a length of less than 2 were discarded for this analysis. The first *n* (length of the generated sequence) proteins were taken from the original sequences to be compared with the generated one, since the latter is usually shorter than the original one. This occurs since the bidirectional LSTM cannot produce a confident set of new proteins, and the generation process stops. Figure 6a shows the Jaccard measure between the real and generated sequences of Bone. In this heat map, intense blue means that the generated sequence contains several proteins that are also in real sequences. The Levenshtein distance is shown in Figure 6b; intense blue means that many operations such as proteins deletion and insertion transform the generated sequence into a real sequence. Figure 6 supports the idea that the generated sequences contain many proteins of the real sequence (intense blue in the Jaccard heat map) and that the proteins in the sequences are in the true positions (light blue in the Levenshtein heat map). Hence, the generated and real sequences match in terms of the proteins and their positions. The plots for the remaining functions are in the Supplementary Material; see Figures S2–S5. Table 5 summarizes the Jaccard measure, the Levenshtein distance, and the length ratio of generated and real sequences grouped by the function of human organs.

The results of Table 5 show that the generated sequences of Bone contain about 50% of the proteins (Jaccard measure) in the original sequence; meanwhile, the proteins in the generated sequences of Motor are about 24% of the proteins contained in the original sequence. Furthermore, the Levenshtein distance is the erroneous relevance forecasted (position in the sequence); it ranges from 12 to 22. For example, the Levenshtein distance of the generated sequence *G* and the real *R* is 7; see the Brachydactyly type D network in Table 6. The first four proteins match in both sequences; however, the five in *G* differ from those in the same position in *R*. *G* can be converted into *R* by (1) inserting “RAB7A” and replacing the proteins in the (2) sixth, (3) seventh, (4) eighth, (5) ninth, (6) tenth, and (7) eleventh positions. In practice, this information determines the cost of finding the true protein sequence, which can help researchers in guiding experimental design, understanding pathogenesis, and finding key points for new therapeutics. Finally, the length ratio is the percentage of the total proteins of the real sequence generated. In general, the bidirectional LSTM produced sequences with a length from 14% to 21% of the original sequences.

**Figure 7:**
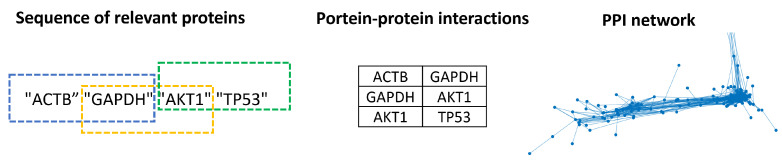
Detection of spurious PPIs in relevant node sequences.

Finally, the sequences of the relevant proteins extracted from PPI networks (by the fractal method) and generated by bidirectional LSTM contained spurious interactions. For example, the first two proteins in a relevant protein sequence could not be directly connected in the network from which it was extracted. Although our approach does not try to identify positive and negative interactions, those contained in the sequences are tested using real accuracy and random accuracy [79]. First, the sequences were fragmented in pairs, as in [76]. Let “ACTB”, “GAPDH”, “AKT1”, and “TP53” be a sequence of relevant nodes; the first PPI is “ACTB” and “GAPDH”, and the second is “GAPDH” and “AKT1”; see the middle of Figure 7 for the resulting PPI. The PPIs of the relevant protein sequences are then tested to determine if they are in the network where it was extracted (an arc between these proteins must exist in the network). Meanwhile, the PPI generated by the bidirectional LSTM was tested to determine whether it belonged to the set of PPI network groups by the function of the human organs used to train the bidirectional LSTM.

Table 7 demonstrated that the mean accuracy (0.949) (see the $Acc_{e}$ column) of extracted protein sequences is similar to that of the generated ones (0.9486) (see $Acc_{g}$). These true PPIs extracted from PPI networks were also learned by the bidirectional LSTM producing a low rate of spurious PPIs. Furthermore, the fractal method extracts a high number of true PPIs, even though it was not designed for this objective. This low number of spurious PPIs is reflected in the high values of random accuracy; see Acc(r) in the generated and extracted columns of Table 7. Random accuracy is the classification rate of the hypothetical random model [79]. For example, if an extremely biased model classifies each current PPI as true, then the number of correct classifications of spurious PPIs will be zero, and the correct classification of true PPIs will equal its number; hence, the random accuracy depends on how balanced the spurious and true PPIs are. In the balanced data (where 50% of PPIs are spurious and 50% are true), our biased model will obtain an accuracy of 0.5, equivalent to a random classification.

## 6. Discussion and Conclusions

This study introduces an approach for generating a relevant protein sequence based on bidirectional LSTM with partial knowledge of true PPIs. The general aim of the framework was to conduct scale-free and fractal analysis to determine the topology of PPI networks. The results demonstrate that a handful of PPI networks are self-similar or fractal, but both cannot coexist (the union of scale-free networks (Table S2) and of fractal networks (Table S3) is empty). The hub repulsion is a feature that causes the emergence of fractality [53,80] but is not the only one. On the other hand, the Barabasi–Albert [81] model generates scale-free networks but not fractal ones. In Kuang et al. [82], the model proposed by Song et al. [53] was extended to conciliate these two approaches. Their results show that the scale-free property and fractality coexist in some networks, with hub attraction and a high clustering coefficient for each box (a property that the Songs networks do not have [83]). This result coincides with the work of Ikeda [84], wherein a network model was proposed to generate fractal and scale-free networks based on a high clustering local property. The PPI networks have hub repulsion, meaning that the most important proteins are not directly linked to others but through proteins with fewer connections generating the fractal property. On the other hand, in less analyzed scale-free PPI networks, the hubs are linked to each other directly, but the non-hub nodes in the boxes are poorly connected, preventing the fractal property from emerging. In summary, research on fractal PPI disease networks should focus on the interactions between the non-hubs of the boxes. However, scale-free PPI disease networks must center on the hubs.

Furthermore, based on these results, the fractal attack was selected over the maximum degree-based method for extracting relevant proteins. The sequences extracted by the fractal method are highly correlated with the resilience (measured by the AURC) of the PPI networks, and the fractal extraction produces an average of 94.9% of true PPI sequences. This remarkable feature is also presented in the sequences generated by the bidirectional LSTM, which reaches approximately 94.8% of true PPIs and is comparable with previous studies [30,76].

The generated PPI sequences contain an average of 39.5% of proteins that are in the original sequence (the Jaccard measure), and the bidirectional LSTM was able to generate about 25 proteins per sequence by only using the extracted sequences obtained by the fractal method. The ratio between the generated and original sequences of proteins was 17%. This means that large sequences were produced with partial PPI information, given that the mean number of proteins in the original sequences is 303.95 (Length(original)=Length(generated)/Length(ratio)). Moreover, these sequences of proteins (that are ordered from high to low relevance) can drive the search for true but unknown PPIs. The results show that the proposed method relies on the sample PPI networks selected to produce the new sequences; thus, it requires careful selection. The results demonstrate that the spurious PPIs in the sequences (extracted and produced) originated from the fractal method, which was only designed to find relevant nodes, such as in [43]. This paves the way toward the creation of an ad hoc algorithm that reduces false PPIs but finds the essential proteins. The automatic generation of PPI sequences can be a powerful tool for understanding biological processes without limitations such as costs, resources, and time.

## Figures and Tables

**Table 1:** Classification of PPI networks by functions of human organs.

Functions of Human Organs	PPI Networks
Immune	38
Metabolism	27
Motor	45
Nerve	67
Bone	79
Endocrine	43
Cardiovascular	27
Brain	45
Generalized	28
Others	232

**Table 2:** The summary of fit of several distribution models to the node degree of PPI networks.

Distribution	Networks	Percentage
power law with cutoff	4	0.84
exponential	163	34.1
exponential or log-normal	30	6.27
log-normal	281	58.78
**total **	**478**	**100**

**Table 3:** The summary of the fit of several models to the minimum number of boxes needed to cover the PPI networks.

Function	Networks	Percentage
delayed exponential	276	57.74
delayed fractal	97	20.29
exponential	11	2.30
exponential or delayed exponential	90	18.83
exponential or fractal	4	0.84
**total**	**478**	**100**

**Table 4:** The MAPE, MAE, RMSE, and $R^{2}adj$ μ(σ) of AURC regression.

	Fractal Method	Degree-Based Method	*t*-Test
MAPE	12.544(0.9169)	13.839(0.916)	*t*(25.2) = 2.394; *p* = 0.024
MAE	0.031(0.005)	0.034(0.002)	*t*(27.020) = 2.762; *p* = 0.01
RMSE	0.039(0.007)	0.043(0.002)	*t*(24.254) = 2.191; *p* = 0.038
$R^{2}adj$	0.806(0.059)	0.776(0.022)	*t*(24.233)=-2.198; *p* = 0.038

**Table 5:** The Jaccard measure, the Levenshtein distance, and the length (ratio) μ(σ) of the function of organ sequences.

Function	Jaccard	Levenshtein	Length (Ratio)	Length (Generated)
Immune	0.444 (0.278)	12.571 (11.698)	0.182 (0.234)	20.21 (16.741)
Metabolism	0.431 ( 0.320)	22.1 (23.921)	0.192 (0.24)	34.80 (30.583)
Motor	0.252 (0.232)	14 (10.863)	0.14 (0.162)	19.30 (17.169)
Nerve	0.324 (0.269)	15.118 (14.447)	0.152 (0.185)	22.04 (19.976)
Bone	0.523 (0.295)	14.952 (18.180)	0.218 (0.24)	31.81 (43.986)
Mean	0.395	15.748	0.177	25.632

**Table 6:** Examples of generated sequences (G) and real ones (R). Only the first eleven proteins are shown to simplify the table.

Network	Type	Sequence										
Brachydactyly type D	*G*	ACTB	ALB	GAPDH	CANX	INS	GNAS	BMP2	STX16	RPS3	CD4	THY1
Brachydactyly type D	*R*	ACTB	ALB	GAPDH	CANX	RAB7A	INS	IHH	IL10	GNAS	HSPA4	BMP2
Multiple congenital anomalies-hypotonia-seizures syndrome	*G*	PIGG	MPPE1	PGAP1	CD59	PIGZ	PIGQ	PIGT	PGAP2	CD55	RER1	TMED10
Multiple congenital anomalies-hypotonia-seizures syndrome	*R*	PIGG	MPPE1	PGAP1	CD59	PIGZ	PIGQ	PIGT	PGAP2	CD55	RER1	TMED10
Autosomal dominant auditory neuropathy 1	*G*	OTOF	MYO6	FMN1	ACTG1	CDH23	MYO7A	DIAPH3	RHOA	DIAPH1	RAC3	RAC2
Autosomal dominant auditory neuropathy 1	*R*	OTOF	MYO6	FMN1	ACTG1	CDH23	MYO7A	DIAPH3	CDC42	RHOA	DIAPH1	RAC3
Charcot–Marie–Tooth disease axonal type 2CC	*G*	SOD1	SYT1	FSCN1	PSEN1	GDAP1	RAB5A	DCTN1	WAS	YARS	KIF5A	SNCA
Charcot–Marie–Tooth disease axonal type 2CC	*R*	SOD1	FSCN1	DCTN1	PSEN1	NOTCH3	NEFL	YARS	KIF5A	DYNC1H1	SNCA	FUS
Diabetes Mellitus RI	*G*	INS	SLC2A2	GCG	HNF4A	PPARG	AKT1	PAX4	ZFAND3	IL6	ADIPOQ	LEP
Diabetes Mellitus RI	*R*	INS	SLC2A2	GCG	HNF4A	ALB	PPARG	GCK	AKT1	PAX4	GLIS3	ZFAND3

**Table 7:** The real (Acc) and random accuracy (Acc(r)) of relevant protein sequences extracted by the fractal method and those generated by bidirectional LSTM. e means extracted by fractal method, g means generated by bidirectional LSTM, and Sp means spurious.

Net	$Acc_{e}$	$Acc{\left(r\right)}_{e}$	$PPI_{e}$	$True_{e}$	$Sp_{e}$	$Acc_{g}$	$Acc{\left(r\right)}_{g}$	$PPI_{g}$	$True_{g}$	$Sp_{g}$
Immune	0.936	0.935	1638	1533	105	0.951	0.949	533	507	26
Metabolism	0.936	0.935	1697	1588	109	0.96	0.958	672	645	27
Motor	0.966	0.965	1642	1586	56	0.915	0.914	601	550	51
Nerve	0.960	0.959	1716	1648	68	0.963	0.962	1067	1028	39
Bone	0.947	0.946	1649	1561	88	0.954	0.953	1895	1808	87

## Data Availability

The data that support the findings of this study are available from the corresponding author upon reasonable request.

## Supplementary Materials

The following supporting information can be downloaded at https://www.mdpi.com/article/10.3390/biology12010140/s1. Table S1: Terms and suffixes employed to query the DISEASES on Cytoscape; Table S2: The ΔAIC of 476 human PPI networks for node degree distribution; the values in bold are those less than 2; Table S3: The ΔAIC of 476 human PPI networks for box covering; the values in bold are those less than 2; Figure S1: The fit of several models for the node degree probability distribution of (a) Endocarditis network and (b) Gilles de la Tourette syndrome network; Figure S2: The (a) Jaccard measure and (b) Levenshtein distance between real and generated sequences of Immune; Figure S3: The (a) Jaccard measure and (b) Levenshtein distance between real and generated sequences of Metabolism; Figure S4: The (a) Jaccard measure and (b) Levenshtein distance between real and generated sequences of Motor; Figure S5: The (a) Jaccard measure and (b) Levenshtein distance between real and generated sequences of Nerve; Figure S6: The box-covering implementation example of (a) brief network. (b) The result of the box number for node two for a size one. (c) The result of the box number for node two for a size two. (d) The box assignment for the network’s six nodes for the box size from one to five.
