# Supplementary material for: Identification of Relevant Protein Interactions with Partial Knowledge: A Complex Network and Deep Learning Approach
Source: Biology (Basel). 2023 Jan 16;12(1):140. doi: 10.3390/biology12010140 (PMC9856098; doi:10.3390/biology12010140)
Supplement: Supplementary file 1 [file biology-12-00140-s001.zip › biology-2066052-supplementary.pdf]

Identification of relevant proteins with partial knowledge of the interaction network

SUPPLEMENTARY MATERIAL

Terms and suffixes of the query

Table S1 shows the terms and suffixes of the query used in Cytoscape 3.9.1 with the plugin DISEASES stringApp to visualise and retrieve networks from the STRING database.

Table S1. Terms and suffixes employed to query the DISEASES on Cytoscape.

Acute	Chol	Idiopathic	Onset
Adeno	Chronic	Igg	Osgood
Agenesis	Colle	Immun	Osteo
Alveo	Colon	Integu	Osteogenesis
Amel	Color	Intestinal	Ovar
Amyl	Corneal	Intra	Pain
Amyo	Cranial	Iso	Para
Anemia	Dementia	Juve	Parkinson
Aneurysm	Dental	Kohler	Parkinson
Angiopathy	Disease	Late	Pneumo
Ankylosing	Disorder	Legg	Peri
Aortic	Dominant	Left	Poly
Apo	Don	Leigh	Prima
Arteritis	Drug	Leukemia	Progress
Astro	Dystrophic	Lipo	Pulmo
Astrocytic	Egg	Light	Renal
Ataxia	Endo	Macular	Res
Auto	Epi	Megaloblastic	Rheuma
Biotin	Epilepsy	Meta	Schi
Blood	Erosion	Mito	Second
Brain	Exos	Mitral	Selec
Breast	Familia	Mono	Stroke
Brugada	Fatty	Monocytic	Synda
Bull	Fibro	Motor	System
Cal	Fibroadenoma	Moya	Third
Capillary	Ganglio	Muscle	Thyro
Carci	Gastro	Multi	Trans
Cardiac	Glaucoma	Myelomonocytic	Tumor
Cardio	Glioma	Myocardial	Type

Cerebellar	Glyco	Myoclonic	Valve
Ceroid	Heart	Myos	Veno
Cervix	Hemo	Neo	Viral
Charcot	Hepat	Nephro	Vitelli
Child	Hipo	Neuro	Werd
Childhood	Hyper	Nose	Wolf

Analysis

The Supplementary Material shows detailed fractal, self-similar analysis based on Akaike information criterion (AIC) [47].

Table S2 shows the model selection of power law Equation (1), power law with a cutoff Equation (2), exponential Equation (3), Weibull Equation (4), and log-normal Equation (5) based on AIC. The ΔAIC_e, ΔAIC_w, ΔAIC_p, ΔAIC_{ln}, ΔAIC_{pc} correspond to exponential, Weibull, power law and power law with a cutoff, respectively. The AIC of each model were not reported in Table S1 for simplicity, but $AIC_i = \Delta AIC_i + AICmin$. The best model is based on ΔAIC=0.

Table S2. The ΔAIC of 476 human PPI networks for node degree distribution, the values in bold are those two less than two.

PPI network	Best model	AIC_{min}	ΔAIC_e	ΔAIC_w	ΔAIC_p	ΔAIC_{ln}	ΔAIC_{pc}
Alzheimer's disease	exponential	-21249.62	**0**	91.3770087	18602.0514	148.037875	154.050653
Amelogenesis imperfecta type 1B	exponential	-943.63476	**0**	15.9792118	649.276115	4.89809515	17.591571
Amelogenesis imperfecta type 1E	exponential	-1315.4469	**0**	3.48727012	1048.08011	3.40092243	12.3700432
Amelogenesis imperfecta type 3	exponential	-729.15649	**0**	4.83761699	282.19913	20.150041	20.8919434
Amyotrophic lateral sclerosis type 2	exponential	-2238.5434	**0**	4.81514817	1348.29249	39.0958494	40.9269595
Amyotrophic lateral sclerosis	exponential	-21075.714	**0**	113.548043	18014.8641	110.463137	171.087173
Anemia	exponential	-22530.515	**0**	151.003391	19778.6838	108.629099	206.876883
Angiodysplasia	exponential	-992.01276	**0**	2.00542311	604.423923	**1.21829564**	15.5199122
Apoptosis	exponential	-8225.9867	**0**	62.7575372	3134.02293	193.912504	175.079847
Arterial calcification of infancy	exponential	-1185.8145	**0**	2.8576997	551.011479	11.1572289	17.7104003
Arts syndrome	exponential	-1005.6228	**0**	6.48549557	17.3884243	**1.03682183**	16.2243105
Autosomal dominant Alport syndrome	exponential	-638.67937	**0**	8.98956978	13.1590769	**1.56102484**	13.4762796
Autosomal dominant Emery-Dreifuss muscular dystrophy 4	exponential	-809.233	**0**	6.94289088	193.325425	8.14166139	15.0708617
Autosomal dominant auditory neuropathy 1	exponential	-1214.378	**0**	4.12451087	291.699248	**1.08523372**	14.6825246
Autosomal dominant cerebellar ataxia, deafness and narcolepsy	exponential	-1131.5807	**0**	6.98901279	799.957837	5.91260626	16.7507326
Autosomal dominant cerebellar ataxia	exponential	-20370.563	**0**	96.2404157	17351.121	38.1070447	135.336939

PPI network	Best model	AIC_{min}	ΔAIC_e	ΔAIC_w	ΔAIC_p	ΔAIC_{ln}	ΔAIC_{pc}
Autosomal dominant cutis laxa	exponential	-1188.372	**0**	6.17548527	836.666984	4.99324924	17.2073057
Autosomal dominant disease	exponential	-21495.597	**0**	47.1860788	17569.38	44.7929011	88.6207294
Autosomal dominant hypocalcemia	exponential	-1389.6225	**0**	9.33065305	1055.9505	6.75954929	19.9320327
Autosomal dominant microcephaly	exponential	-645.40613	**0**	2.06259484	473.934309	7.58357785	16.186681
Autosomal dominant non-syndromic intellectual disability	exponential	-9631.1388	**0**	28.675519	7633.76869	13.1859919	86.2831998
Blood group incompatibility	exponential	-755.98898	**0**	3.3836196	576.144094	7.77815541	14.3279921
Blood platelet disease	exponential	-21862.799	**0**	27.3993205	19717.4447	8.31978743	60.7186478
Borjeson–Forssman–Lehmann syndrome	exponential	-1492.9067	**0**	31.7418129	923.016032	19.7128124	40.481604
Brachydactyly type C	exponential	-598.331	**0**	4.97772221	327.679548	**1.85236131**	11.9370304
Brachydactyly type D	exponential	-1672.9708	**0**	14.8630765	468.907067	40.8828684	58.4579538
Brain ischemic RI	exponential	-967.59821	**0**	103.171038	500.078577	88.8614106	64.1855948
Bruck syndrome	exponential	-1500.3496	**0**	2.02478203	1111.1113	6.92726389	21.8282944
COVID-19	exponential	-745.13353	**0**	30.7121464	398.215667	3.37926781	24.540672
Carbohydrate metabolic disorder	exponential	-21589.65	**0**	97.0003166	18710.1505	265.809128	184.307037
Cardiomyopathy	exponential	-20447.657	**0**	80.1511304	17929.5005	92.2200463	132.138481
Cenani–Lenz syndactyly syndrome	exponential	-760.58688	**0**	4.22568252	604.504913	8.19240968	17.2067201
Cervix small cell carcinoma	exponential	-217.17448	**0**	4.77232497	165.882507	4.73492694	8.15720484
Cervix uteri carcinoma in situ	exponential	-2932.7901	**0**	20.4532897	1648.89077	9.03661647	59.3955087
Charcot–Marie–Tooth disease axonal type 2CC	exponential	-520.43541	**0**	9.08660309	37.4051541	6.49615844	15.2564719
Charcot–Marie–Tooth disease dominant intermediate B	exponential	-1902.6619	**0**	40.4555575	1403.01457	25.2443074	44.9731437
Charcot–Marie–Tooth disease dominant intermediate E	exponential	-721.24311	**0**	19.3657314	354.152352	**1.81991838**	19.127671
Charcot–Marie–Tooth disease type 1	exponential	-625.27387	**0**	22.1213433	374.806881	11.4787651	21.6981366
Charcot–Marie–Tooth disease type 1A	exponential	-4960.2244	**0**	2.06643191	3849.48949	8.00390496	33.2217268
Charcot–Marie–Tooth disease type 1D	exponential	-1071.5809	**0**	6.14944101	399.534663	17.1447238	17.9058918
Charcot–Marie–Tooth disease type 1E	exponential	-1552.1912	**0**	18.466977	1096.98226	11.9841641	26.1890196
Charcot–Marie–Tooth disease type 1F	exponential	-1555.3308	**0**	14.2766302	916.699347	3.02570345	23.2317074
Charcot–Marie–Tooth disease type 2A1	exponential	-834.41584	**0**	16.8783271	281.339442	4.42321375	18.3432064
Charcot–Marie–Tooth disease type 2B2	exponential	-1138.7077	**0**	33.2733197	705.771947	21.5966352	36.8379848
Charcot–Marie–Tooth disease type 2E	exponential	-1783.8479	**0**	12.3798758	1285.6458	8.62381326	23.8453805
Charcot–Marie–Tooth disease type 2I	exponential	-774.53109	**0**	5.47787451	365.1569	5.35236389	12.7271787
Charcot–Marie–Tooth disease type 3	exponential	-1480.6052	**0**	3.5718079	836.650439	11.3440636	24.0629532
Charcot–Marie–Tooth disease type 4B2	exponential	-2320.3021	**0**	12.6615393	1773.92722	3.48687114	23.4531288
Charcot–Marie–Tooth disease type 4D	exponential	-1392.8102	**0**	4.53069597	1141.84798	7.98219466	22.056491

PPI network	Best model	AIC_{min}	ΔAIC_e	ΔAIC_w	ΔAIC_p	ΔAIC_{ln}	ΔAIC_{pc}
Charcot–Marie–Tooth disease type 4E	exponential	-903.86647	**0**	6.99673614	440.763442	8.51338443	15.344149
Charcot–Marie–Tooth disease type 4F	exponential	-1345.7907	**0**	11.5512258	703.138445	13.8511087	22.6810902
Charcot–Marie–Tooth disease type X	exponential	-2803.0142	**0**	36.0792513	1871.62699	**0.09606681**	41.955155
Childhood acute myeloid leukemia	exponential	-4027.195	**0**	34.3932654	3448.24798	33.1208052	92.6941253
Chromosome 2q37 deletion syndrome	exponential	-1858.2731	**0**	2.09905304	620.839063	39.6913768	30.6145125
Colon Cancer RI	exponential	-963.37509	**0**	132.200648	329.58276	99.5803632	71.3261293
Colon cancer	exponential	-20629.533	**0**	106.761527	16938.633	212.281906	183.60741
Colon carcinoma	exponential	-20607.594	**0**	58.3581927	13732.8979	**0.03021042**	98.5965882
Colon mucinous adenocarcinoma	exponential	-558.01403	**0**	15.2428221	427.169721	19.4178254	21.3377315
Colonic disease	exponential	-20780.448	**0**	106.250911	17566.052	191.471226	175.49826
Color blindness	exponential	-4123.7025	**0**	8.13318988	2085.10547	44.7729574	57.0951944
Colorectal adenoma	exponential	-15249.237	**0**	73.0202197	13971.3356	23.684883	128.759137
Congenital contractural arachnodactyly	exponential	-1988.4998	**0**	8.25549343	1155.57121	36.785921	58.6870614
Cranial nerve disease	exponential	-20946.893	**0**	48.0412536	18999.3457	122.348815	97.7835966
Crohn colitis	exponential	-2289.9453	**0**	10.6581704	1788.80802	5.29220786	34.2165314
Degenerative myopia	exponential	-1429.1382	**0**	4.64296451	772.493318	8.23794831	23.7967217
Disease of metabolism	exponential	-21464.683	**0**	70.018206	16100.0139	218.472604	136.234491
Disorder of sexual development	exponential	-18999.284	**0**	12.2907713	16894.1358	47.9982306	46.9843026
Donnai-Barrow syndrome	exponential	-1747.8019	**0**	39.0731397	1134.24186	53.2496599	62.4049253
Drug psychosis	exponential	-1229.5162	**0**	2.02762819	607.155572	12.351706	20.0192652
Drug-induced lupus erythematosus	exponential	-699.89856	**0**	3.10986731	538.217473	4.15598448	14.0869529
Drug-induced mental disorder	exponential	-1246.5382	**0**	2.00324434	623.732773	14.0397112	21.0232692
Dyscalculia	exponential	-1481.5177	**0**	5.79143373	1238.73623	11.2330624	29.1287101
EPICATECHIN 2	exponential	-933.52043	**0**	56.1306138	429.95345	10.7512937	39.0654976
Endocarditis	exponential	-10045.001	**0**	49.5265229	9003.75048	43.2803847	92.9496627
Esotropia - 1	exponential	-3792.4623	**0**	49.5606248	2796.66777	13.2264691	67.5360131
Gilles de la Tourette syndrome	exponential	-7048.471	**0**	26.3064606	6026.3501	72.5773339	93.0607903
Glucose metabolism disease	exponential	-21564.155	**0**	93.358048	19006.9382	237.110405	169.98573
Glycogen storage disease IX	exponential	-768.86337	**0**	3.63702645	616.617021	2.6074804	16.7847848
Hereditary spastic paraplegia 18	exponential	-953.2061	**0**	2.0652534	236.640619	8.98229124	12.292799
Holocarboxylase synthetase deficiency	exponential	-619.46963	**0**	34.6477998	274.10504	16.8889323	31.7094269
Huntington's disease	exponential	-20660.739	**0**	166.834179	17849.0688	213.274493	241.676314
Iimmunodeficiency 60	exponential	-554.09471	**0**	3.88082056	312.337216	**1.46505656**	12.422838
Immunodeficiency 50	exponential	-503.54987	**0**	15.06506	379.652422	14.4735628	19.0333376

PPI network	Best model	AIC_{min}	ΔAIC_{e}	ΔAIC_{w}	ΔAIC_{p}	ΔAIC_{ln}	ΔAIC_{pc}
Intrahepatic cholestasis	exponential	-6888.4201	**0**	3.83514837	5734.76702	3.31843268	31.9091013
Ischiocoxopodopatellar syndrome	exponential	-553.11924	**0**	3.10711476	427.20487	15.1695691	19.9914681
Isolated growth hormone deficiency type IB	exponential	-629.45751	**0**	38.5565369	452.612506	30.0443439	38.6802807
Kidney failure	exponential	-21226.154	**0**	28.5302761	19085.6233	38.2158821	68.2151988
Legg–Calve–Perthes disease	exponential	-1385.3962	**0**	2.30094359	1087.15998	5.92876947	27.2266604
Lipid metabolism disorder	exponential	-20816.892	**0**	54.2446709	16203.3031	124.189478	109.086421
Lleukemia	exponential	-21341.819	**0**	115.097177	17092.3988	106.39015	173.023148
Llipid metabolism disorder	exponential	-20814.454	**0**	53.9402902	16201.1288	124.947081	108.782434
Lysosomal storage disease	exponential	-21091.061	**0**	45.994845	19204.1472	76.8616302	92.9656698
Meckel's diverticulum	exponential	-2100.5546	**0**	7.58911289	1705.93197	3.09437164	38.9166067
Melnick–Needles syndrome	exponential	-1295.1784	**0**	10.7265821	1033.29214	54.5838512	47.2238463
Mitochondrial complex I deficiency	exponential	-3705.1801	**0**	29.0965617	622.781544	58.1191513	81.0566598
Motor neuritis	exponential	-1017.579	**0**	10.5078271	490.138067	7.91524703	18.1940193
Motor neuron disease	exponential	-21134.909	**0**	132.796021	19768.1876	144.95614	196.178231
Moyamoya disease	exponential	-3225.4597	**0**	38.1176535	1020.96332	78.5198478	98.6832387
Multiple epiphyseal dysplasia	exponential	-2513.7233	**0**	6.63574776	2072.06686	7.66898785	47.0563143
Multiple intestinal atresia	exponential	-961.29629	**0**	8.51931209	664.72789	**1.5136314**	16.9912812
Multiple sclerosis	exponential	-21476.966	**0**	59.8765045	19763.32	137.671615	115.008436
Multiple symmetric lipomatosis	exponential	-657.89955	**0**	3.34327624	500.853835	2.03752427	8.08019841
Myocardial infarction RI	exponential	-896.44215	**0**	36.3468858	542.112245	13.8383252	31.5875545
Myosin	exponential	-935.77589	**0**	64.2186229	186.379178	28.5385096	46.5384445
Nephronophthisis 1	exponential	-1187.2614	**0**	3.09932077	168.119514	24.6855911	44.3170971
Nephronophthisis 4	exponential	-1391.654	**0**	2.49034175	469.795054	15.5029038	31.2106258
Neuroacanthocytosis	exponential	-2356.2049	**0**	5.23078574	1523.93418	3.48061845	26.280753
Neuronal ceroid lipofuscinosis 8 northern epilepsy variant	exponential	-701.9271	**0**	7.43658702	419.212573	27.8142634	29.9170062
Neuropathy	exponential	-21468	**0**	89.2897466	19081.8527	206.088693	159.213995
Nose disease	exponential	-20301.95	**0**	62.1822489	18797.129	35.7244351	102.980529
Obesity	exponential	-21206.401	**0**	50.4559523	16543.374	138.898927	104.484057
Ollier disease	exponential	-3079.0441	**0**	16.0603469	2465.83423	**1.31992836**	50.8274784
Optic nerve disease	exponential	-20855.401	**0**	36.2673173	18737.8308	87.4625662	78.1033937
Pancreas disease	exponential	-20661.664	**0**	71.3481771	15908.537	74.6373042	125.259652
Paranoid schizophrenia	exponential	-2577.3472	**0**	2.6900607	1738.70334	14.1169855	27.5563964
Phosphoglycerate kinase 1 deficiency	exponential	-989.07979	**0**	33.8796956	667.98887	13.6480166	33.8883439
Pneumonia	exponential	-21602.714	**0**	7.38423855	19892.5594	3.53793125	34.5625343

**PPI network**	**Best model**	**AIC_{min}**	**ΔAIC_e**	**ΔAIC_w**	**ΔAIC_p**	**ΔAIC_{ln}**	**ΔAIC_{pc}**
Polycystic kidney disease	exponential	-19746.887	**0**	9.60532651	16576.6242	14.487323	41.1763412
Pool Akt AMPK mTORparamat	exponential	-240.10817	**0**	36.6810063	208.103508	45.9884715	36.4688329
Primary amebic meningoencephalitis	exponential	-888.97926	**0**	6.49353975	752.62281	3.99031433	13.6310697
Primary angle-closure glaucoma	exponential	-3273.8457	**0**	11.1501993	1528.22312	29.7582753	46.2202097
Primary cutaneous amyloidosis	exponential	-2769.1988	**0**	21.3171253	662.253756	52.3080326	63.8139821
Primary failure of tooth eruption	exponential	-534.95841	**0**	24.289439	341.326847	10.7834593	22.4073329
Primary hyperaldosteronism	exponential	-9351.6637	**0**	25.2546747	8234.10461	9.94594385	44.9122682
Primary hypomagnesemia	exponential	-1217.7176	**0**	19.0871142	760.444801	9.16809377	27.2897272
Progressive bulbar palsy	exponential	-1306.0656	**0**	2.62848747	442.045333	21.8778941	22.1718638
Progressive muscular atrophy	exponential	-2254.9045	**0**	4.95646603	1388.80117	14.8047744	25.4750027
Progressive osseous heteroplasia	exponential	-1144.2169	**0**	2.94084853	961.833724	10.6237012	15.1888494
Renal adenoma	exponential	-1059.7527	**0**	2.94426086	841.440696	**0.36738808**	11.5588252
Renal coloboma syndrome	exponential	-1857.0185	**0**	6.43954933	1202.4508	6.49925196	23.8162879
Renpenning syndrome	exponential	-831.53311	**0**	37.682171	603.788703	27.22509	39.7003235
Sarcoma	exponential	-21676.821	**0**	111.261036	16997.6094	82.48282	167.462604
Scarlet fever	exponential	-528.70254	**0**	2.27134285	80.5785522	**1.45539598**	9.67184973
Schizophrenia	exponential	-19726.574	**0**	47.0479956	18166.8664	45.8766374	88.6713108
Schwartz–Jampel syndrome 1	exponential	-513.9037	**0**	7.49605445	215.18697	13.5744097	18.7653579
Secondary Parkinson disease	exponential	-1600.3266	**0**	2.34087461	1102.51018	9.75199601	19.5952915
Segmental dystonia	exponential	-1152.0668	**0**	17.0856863	861.510948	16.6679367	24.7329071
Selective IgA deficiency disease	exponential	-988.60954	**0**	3.61826359	807.039346	21.1787232	22.2060069
Sjogren–Larsson syndrome	exponential	-1082.1555	**0**	6.19330057	483.92185	10.3567091	18.4833945
Small intestine adenocarcinoma	exponential	-1249.3123	**0**	7.68088857	926.131143	19.3657	40.5906902
Small intestine neuroendocrine neoplasm	exponential	-771.98095	**0**	2.04068923	539.565998	19.7193132	22.9169147
Syndrome	exponential	-21526.469	**0**	53.0509148	19234.1282	27.5262385	91.015386
Syndromic X-linked intellectual disability	exponential	-6481.8156	**0**	30.2244755	4389.16034	14.4687814	60.2490472
Syndromic microphthalmia	exponential	-3468.8891	**0**	2.17804649	2755.91365	10.3674301	37.1823571
Thyroid adenoma	exponential	-7572.7207	**0**	66.8262574	6039.22636	86.435725	123.123911
Thyroid gland adenocarcinoma	exponential	-7545.4863	**0**	60.7924657	5419.3368	16.7423857	117.212937
Thyroid gland follicular carcinoma	exponential	-7512.6687	**0**	61.7736925	5402.82215	20.165353	118.471713
Tooth agenesis	exponential	-5915.9222	**0**	32.5854893	3520.01915	57.6753423	103.690044
Trichohepatoenteric syndrome	exponential	-1674.0146	**0**	13.9292758	954.918185	4.92995388	25.1896764
Uterus carcinoma in situ	exponential	-2932.7901	**0**	20.4532897	1648.89077	9.03661647	59.3955087
Vici syndrome	exponential	-2857.4917	**0**	9.35423647	2032.69505	8.01325216	39.8992995

PPI network	Best model	AIC_{min}	ΔAIC_e	ΔAIC_w	ΔAIC_p	ΔAIC_{ln}	ΔAIC_{pc}
X-linked intellectual disability-psychosis-macroorchidism syndrome	exponential	-634.3422	**0**	9.37353268	322.502001	17.2098957	18.564417
Aachondroplasia	exponential	-6039.1955	**0**	29.2572539	4320.79062	15.365139	70.168096
Aacute hemorrhagic conjunctivitis	exponential	-1175.7212	**0**	4.43942807	579.648286	8.95348194	29.8681456
Aautosomal dominant osteopetrosis 2	exponential	-934.34298	**0**	11.0444896	510.951361	19.5884912	26.7511924
Autosomal recessive osteopetrosis 2	exponential	-903.01954	**0**	3.43338939	717.699343	7.80436568	16.3641696
Autosomal recessive osteopetrosis 3	exponential	-907.00408	**0**	15.1785516	523.890059	13.4935394	23.7869814
Blood coagulation disease	exponential	-903.15442	**0**	59.0545673	313.433536	14.8722088	41.1934995
Cleft lip-palate-ectodermal dysplasia syndrome	exponential	-518.23248	**0**	7.7997519	389.55279	12.1560562	17.7040502
Endogenous depression	exponential	-16622.552	**0**	84.5007569	14158.5086	163.696208	173.875078
Gnathodiaphyseal dysplasia	exponential	-1172.3857	**0**	13.72803	801.909809	27.1036817	28.2562526
Hepatitis B	exponential	-19970.149	**0**	51.7264973	18187.194	6.74180418	87.7024
Hepatitis C	exponential	-19590.321	**0**	33.8367084	17339.1432	**1.80761669**	66.7135904
Hepatitis C2	exponential	-19590.039	**0**	33.7410328	17338.9771	**1.48447558**	66.5449776
Hepatitis D	exponential	-1964.6517	**0**	10.9565649	708.761777	18.0110942	44.7463817
Hepatitis	exponential	-20939.85	**0**	114.758393	14583.1281	262.110237	197.898079
Lattice corneal dystrophy	exponential	-731.71426	**0**	3.674037	511.268424	11.8443293	16.9803578
Microcephalic osteodysplastic primordial dwarfism type I	exponential	-2325.318	**0**	6.42150709	1877.32631	14.1017139	39.0385898
Osteochondrodysplasia	exponential	-19985.104	**0**	5.72740637	18007.4749	52.4767434	40.8654499
Osteochondrosis	exponential	-3789.5493	**0**	54.2151937	561.439594	91.2617459	135.585623
Osteogenesis imperfecta type 2	exponential	-500.41059	**0**	5.11088789	90.9180345	8.41232865	11.8255672
Osteogenesis imperfecta type 5	exponential	-635.34332	**0**	4.06653436	490.642399	6.0885782	15.8727683
Osteopathia striata with cranial sclerosis	exponential	-904.86395	**0**	2.41724417	183.539923	14.3254585	23.9745571
Syndactyly type 4	exponential	-722.77316	**0**	12.4614435	504.500826	2.77771898	14.2570557
Synpolydactyly	exponential	-2541.61	**0**	2.44043073	1837.78582	6.23647571	35.9862032
Viral infectious disease	exponential	-21811.285	**0**	59.8846893	19915.6734	21.4656994	102.168198
A amyloidosis	log-normal	-1161.2417	28.9010356	33.1887921	773.928547	**0**	36.0908685
Adenoma 2000	log-normal	-19874.178	106.003677	112.800868	15919.7181	**0**	134.779576
Adrenal adenoma	log-normal	-5017.2512	37.0159346	39.1218186	4329.05761	**0**	57.0052117
Adult acute lymphocytic leukemia	log-normal	-9485.5374	122.370268	133.598115	7840.41307	**0**	164.823466
Alcohol dependence	log-normal	-19693.82	135.400035	141.007897	17676.9211	**0**	173.862768
Alpers–Huttenlocher syndrome	log-normal	-3596.8198	45.8236299	52.4791358	2731.17175	**0**	63.031701
Alveolar echinococcosis	log-normal	-1894.7104	**1.09712612**	9.44904486	1448.98454	**0**	30.6543396
Amelogenesis imperfecta type 1G	log-normal	-19102.974	102.312511	115.046767	13858.57	**0**	137.520998

PPI network	Best model	AIC_{min}	ΔAIC_e	ΔAIC_w	ΔAIC_p	ΔAIC_{ln}	ΔAIC_{pc}
Amyotrophic lateral sclerosis type 4	log-normal	-4910.3548	71.7471521	80.4138128	4089.82283	**0**	91.1453881
Aortic disease	log-normal	-18869.641	113.054993	115.219484	16669.8068	**0**	137.486377
Apparent mineralocorticoid excess syndrome	log-normal	-803.28721	4.11482518	17.5558038	561.44701	**0**	18.2885537
Arthritis	log-normal	-21558.432	95.4018675	99.6655255	19784.835	**0**	121.531548
Asthma	log-normal	-21381.779	39.6723978	45.3130245	19704.1108	**0**	69.6423929
Autosomal dominant Emery–Dreifuss muscular dystrophy 2	log-normal	-2111.2198	22.2337052	29.1340371	1641.32873	**0**	39.3042439
Autosomal dominant centronuclear myopathy	log-normal	-2266.6823	18.4834228	32.2768022	1269.38527	**0**	42.7000363
Autosomal dominant distal hereditary motor neuronopathy	log-normal	-3757.4508	28.8961355	40.6117198	2929.97941	**0**	57.7072216
Autosomal dominant familial periodic fever	log-normal	-4225.5565	63.1483671	66.560567	3150.27044	**0**	81.3881719
Autosomal dominant hypophosphatemic rickets	log-normal	-1694.6684	7.82450324	11.2248194	1098.76009	**0**	18.8144118
Autosomal dominant limb-girdle muscular dystrophy type 1	log-normal	-1786.8818	12.8259292	30.5430007	1330.86498	**0**	32.5662335
Autosomal dominant limb-girdle muscular dystrophy	log-normal	-2265.4391	24.5874927	41.5774698	1695.87282	**0**	44.5687831
Autosomal dominant nocturnal frontal lobe epilepsy	log-normal	-2743.4139	22.0292271	24.8674441	1862.77492	**0**	48.9793148
Autosomal dominant nonsyndromic deafness 1	log-normal	-1668.5398	6.12784151	13.4681816	617.568024	**0**	20.7933561
Autosomal dominant nonsyndromic deafness 10	log-normal	-1074.0962	7.2699643	13.0250281	781.29454	**0**	18.2646513
Autosomal dominant nonsyndromic deafness	log-normal	-4894.1705	**0.32543706**	3.77027959	4109.71004	**0**	35.6928719
Autosomal dominant polycystic kidney disease	log-normal	-14469.577	53.4696108	57.0244987	11170.6311	**0**	82.4632495
Bile duct disease	log-normal	-20052.316	121.224016	128.551398	14267.7952	**0**	149.15473
Blood protein disease	log-normal	-15792.249	170.381294	186.912736	13762.6003	**0**	219.189067
Brachydactyly type A1	log-normal	-981.72602	9.53776298	12.769222	730.026483	**0**	18.4734656
Brachydactyly type A2	log-normal	-1400.4927	6.70156293	11.8770945	1079.617	**0**	16.5105405
Brachydactyly type B1	log-normal	-1061.8732	23.9106325	27.1666913	343.68085	**0**	30.75127
Brachydactyly type B2	log-normal	-762.58429	3.91938737	6.15825591	575.25705	**0**	13.9975576
Brachydactyly-syndactyly syndrome	log-normal	-646.04109	8.02559543	20.41405	63.2597097	**0**	19.9558945
Breast secretory carcinoma	log-normal	-695.35608	5.93343098	8.55676516	481.594013	**0**	23.3343757
Brugada syndrome	log-normal	-4677.631	56.6305583	59.2298132	3147.74782	**0**	77.7534738
Burkitt lymphoma	log-normal	-20189.771	213.405625	236.098065	16824.2519	**0**	249.45044
Buschke–Ollendorff syndrome	log-normal	-1247.6215	12.3419609	24.8891817	934.686043	**0**	28.6023544
CHIME syndrome	log-normal	-1116.5261	**0.15676342**	10.9752555	843.448925	**0**	16.5926374
CINCA syndrome	log-normal	-4316.6362	65.0879341	67.2298392	3198.99616	**0**	77.9174027
Camurati–Engelmann disease	log-normal	-1620.8289	21.2250067	23.2260129	1225.15362	**0**	35.7142864

PPI network	Best model	AIC_{min}	ΔAIC_e	ΔAIC_w	ΔAIC_p	ΔAIC_{ln}	ΔAIC_{pc}
Cancer	log-normal	-21254.958	98.133808	101.767979	17313.1712	**0**	123.512966
Cardiac arrest	log-normal	-14898.333	154.857165	160.277825	12362.1251	**0**	176.938673
Cardiovascular cancer	log-normal	-11205.669	148.812851	150.821533	9323.97255	**0**	164.289147
Carney–Stratakis syndrome	log-normal	-900.47586	6.2199206	15.5273977	495.823739	**0**	17.5748652
Carotid artery disease	log-normal	-7831.2209	90.7999058	94.6456284	5562.91706	**0**	111.798077
Cecal disease	log-normal	-4834.645	71.5024555	79.9028812	3843.22744	**0**	103.7455
Cervical cancer	log-normal	-18553.296	204.768828	219.454507	16960.2274	**0**	232.723085
Cervix carcinoma	log-normal	-17123.809	128.169455	191.089478	13126.0484	**0**	257.009913
Cervix disease	log-normal	-18571.916	188.628002	205.338761	17032.3337	**0**	219.080911
Charcot–Marie–Tooth disease X-linked dominant 1	log-normal	-1680.9462	14.262319	26.7223623	1133.30855	**0**	34.0973047
Charcot–Marie–Tooth disease axonal type 2L	log-normal	-1795.6939	14.7428788	27.5464581	1341.78591	**0**	35.4351394
Charcot–Marie–Tooth disease axonal type 2N	log-normal	-1264.8911	10.2160805	13.2156026	961.014456	**0**	32.9608447
Charcot–Marie–Tooth disease axonal type 2P	log-normal	-1420.0555	3.33620979	18.3564669	1082.78547	**0**	23.3705981
Charcot–Marie–Tooth disease axonal type 2S	log-normal	-973.94203	2.00098788	9.81804915	633.849872	**0**	16.7470373
Charcot–Marie–Tooth disease dominant intermediate C	log-normal	-1362.7766	**0.34559773**	2.48494113	271.331549	**0**	23.444956
Charcot–Marie–Tooth disease recessive intermediate B	log-normal	-901.76162	11.882223	15.9674154	614.260988	**0**	20.8850426
Charcot–Marie–Tooth disease type 1B	log-normal	-2069.7144	16.1717448	24.1200139	1624.7448	**0**	34.4573592
Charcot–Marie–Tooth disease type 1C	log-normal	-1800.127	2.8875244	15.8001034	1409.54846	**0**	22.8491547
Charcot–Marie–Tooth disease type 2	log-normal	-9269.7305	53.8829777	93.8593097	7671.98087	**0**	109.63314
Charcot–Marie–Tooth disease type 2A2A	log-normal	-711.90811	7.58713819	15.0259293	486.670329	**0**	17.8395491
Charcot–Marie–Tooth disease type 2A2B	log-normal	-772.46689	**1.64874144**	16.2138381	509.395186	**0**	17.1823024
Charcot–Marie–Tooth disease type 2B	log-normal	-4574.4073	32.6941863	59.1712864	3593.7974	**0**	65.4809427
Charcot–Marie–Tooth disease type 2B1	log-normal	-1733.249	10.9066361	21.2490998	1170.18693	**0**	32.7014825
Charcot–Marie–Tooth disease type 2D	log-normal	-2513.1908	28.4728431	37.2975355	1885.72492	**0**	47.1647254
Charcot–Marie–Tooth disease type 4	log-normal	-8499.5564	36.1188346	50.6807377	7232.89389	**0**	72.8306621
Charcot–Marie–Tooth disease type 4A	log-normal	-2021.5598	**1.74502114**	14.3141263	1575.82721	**0**	22.5155358
Charcot–Marie–Tooth disease type 4B1	log-normal	-2656.5956	27.076766	43.1286852	2011.82946	**0**	49.0843298
Charcot–Marie–Tooth disease type 4B3	log-normal	-1873.5302	**1.30950009**	9.63832007	1492.51574	**0**	22.0399308
Charcot–Marie–Tooth disease type 4C	log-normal	-2185.155	7.10927317	24.438794	1135.96891	**0**	32.5452288
Charcot–Marie–Tooth disease type 4G	log-normal	-820.19501	**0.58009871**	7.11937418	402.490324	**0**	13.3910566
Charcot–Marie–Tooth disease type 4H	log-normal	-1909.3184	11.6325744	20.7672695	1108.15372	**0**	32.6438752
Charcot–Marie–Tooth disease type 4J	log-normal	-3557.3648	35.7700351	48.9454323	2817.75418	**0**	57.1413134
Charcot–Marie–Tooth disease type 4K	log-normal	-1116.9498	2.58259845	22.3991536	195.942683	**0**	22.9669414

PPI network	Best model	AIC_{min}	ΔAIC_e	ΔAIC_w	ΔAIC_p	ΔAIC_{ln}	ΔAIC_{pc}
Cholesterol embolism	log-normal	-887.42488	4.39910622	11.6361475	591.676676	**0**	14.6052963
Chronic obstructive pulmonary disease	log-normal	-20459.162	118.277476	124.547631	18683.2108	**0**	147.647476
Chronic progressive external ophthalmoplegia	log-normal	-7337.1459	46.7080355	57.0182648	5956.4648	**0**	73.404927
Cocaine abuse	log-normal	-4786.0825	74.8392607	77.2706478	2844.19284	**0**	92.4758469
Collecting duct carcinoma	log-normal	-2326.7641	21.5130453	23.776787	1707.23907	**0**	39.7023511
Coloboma	log-normal	-12447.279	38.9885509	44.7090666	9793.31617	**0**	77.7146181
Colon adenocarcinoma	log-normal	-18668.629	241.710242	243.977648	15683.7724	**0**	264.269452
Colon adenoma	log-normal	-7760.3704	15.8226507	48.0026975	6755.5018	**0**	85.8688807
Colonic benign neoplasm	log-normal	-12158.732	128.377693	138.15503	9587.24936	**0**	163.434415
Colorectal adenocarcinoma	log-normal	-18956.459	182.22841	193.969545	15629.5046	**0**	223.504511
Colorectal cancer	log-normal	-20011.782	86.6596619	123.62359	16539.0377	**0**	150.541819
Colorectal carcinoma	log-normal	-20269.592	85.1064547	129.34132	16727.75	**0**	158.014113
Congenital adrenal hyperplasia	log-normal	-3601.4176	7.64746959	16.8936252	3086.50725	**0**	29.2365581
Connective tissue disease	log-normal	-21597.797	24.504484	32.4245642	19472.0429	**0**	54.7601975
Cowden syndrome	log-normal	-5811.7436	63.4310256	65.670053	5012.1738	**0**	77.7967031
DNA ligase IV deficiency	log-normal	-1873.1878	37.2470633	39.2701005	1211.0477	**0**	46.967822
Degenerative disc disease	log-normal	-6536.3317	41.0850882	62.8406052	5450.53467	**0**	96.1265347
Dental pulp disease	log-normal	-3229.39	59.030405	61.642611	2347.19102	**0**	74.8083025
Diabetes Mellitus RI	log-normal	-756.19715	11.7332438	35.9264738	453.039661	**0**	29.414226
Drug allergy	log-normal	-2728.9905	49.2278385	56.9269204	2155.67877	**0**	58.8290062
Drug dependence	log-normal	-7333.1846	16.5296108	31.7007656	5690.43647	**0**	85.445909
Drug-induced hepatitis	log-normal	-1560.1896	15.4773006	23.9331689	1134.214	**0**	27.0464338
Duodenum adenocarcinoma	log-normal	-1399.4584	**0.40253695**	3.97831424	1004.61083	**0**	21.6351624
Egg allergy	log-normal	-1764.2167	22.9523771	25.0612151	1243.55836	**0**	34.225638
Factor VIII deficiency	log-normal	-1926.5043	19.7167184	22.2404184	1493.84878	**0**	30.102609
Factor XI deficiency	log-normal	-1491.578	35.1122346	38.9205112	1085.86243	**0**	43.402971
Familial medullary thyroid carcinoma	log-normal	-1227.3133	14.8474675	17.4752748	613.529744	**0**	25.8533038
Fatty liver disease	log-normal	-20379.93	41.3562655	66.0960165	16408.8996	**0**	97.2456266
Fibrodysplasia ossificans progressiva	log-normal	-3633.2816	57.6430422	70.3584618	2738.4162	**0**	70.6994143
Frontotemporal dementia	log-normal	-19819.901	94.2145333	132.391994	16860.6952	**0**	158.376743
GRACILE syndrome	log-normal	-2553.1256	58.4115485	60.9362618	1800.8009	**0**	68.9986512
Gastrointestinal tuberculosis	log-normal	-1017.9112	17.6326804	19.8051731	635.832533	**0**	27.4982681
Glycogen metabolism disorder	log-normal	-15131.549	53.6354305	72.5384805	12918.7344	**0**	95.9733294
Glycogen storage disease II	log-normal	-4455.1369	39.9800888	65.1038415	3414.2355	**0**	71.7092974

PPI network	Best model	AIC_{min}	ΔAIC_e	ΔAIC_w	ΔAIC_p	ΔAIC_{ln}	ΔAIC_{pc}
Glycogen storage disease V	log-normal	-3027.8897	29.9288462	46.2684435	2256.92256	**0**	53.1813099
Glycogen storage disease VI	log-normal	-1267.389	7.58692081	19.0267831	906.045147	**0**	23.0498515
Glycogen storage disease VII	log-normal	-1389.5677	9.05039593	32.2666746	823.115122	**0**	31.8567917
Hair disease	log-normal	-20992.517	47.7677855	65.2544592	19153.4362	**0**	93.3328884
Heart aneurysm	log-normal	-1757.1834	10.8731945	14.4846868	1347.3654	**0**	29.9370851
Hematologic cancer	log-normal	-21563.538	122.283231	182.694737	16820.466	**0**	207.42152
Hemophilia B	log-normal	-2678.3314	16.7181875	20.6364271	2212.63013	**0**	40.9039328
Hepatocellular adenoma	log-normal	-4823.5028	77.4132434	80.0174099	3957.42892	**0**	94.6058456
Hereditary multiple exostoses	log-normal	-1925.6538	30.0460314	34.4689355	1296.42403	**0**	42.3087813
Huntington's disease like	log-normal	-639.14416	9.88163613	18.6256922	425.30776	**0**	18.969219
Hyper IgM syndrome	log-normal	-6436.183	91.6382146	111.944771	5160.0687	**0**	111.623474
Hyperprolactinemia	log-normal	-3797.4892	63.9592248	79.2680728	2982.11368	**0**	80.3539991
Hypertension	log-normal	-21022.059	6.86121123	31.5912175	18265.2382	**0**	66.5884283
Hyperuricemia	log-normal	-7947.0588	71.3474684	74.2990201	6537.36278	**0**	94.7969942
Hypothyroidism	log-normal	-20396.587	91.5922191	93.5925216	18064.671	**0**	120.889661
IgA glomerulonephritis	log-normal	-10282.758	45.3883065	69.7664952	8785.02586	**0**	102.693293
IgG4-related disease	log-normal	-2499.2961	41.3527175	43.8557224	1856.4967	**0**	55.4958509
Iinterstitial nephritis	log-normal	-5892.4394	56.1191254	59.6702329	4529.66984	**0**	75.034719
Iisolated growth hormone deficiency	log-normal	-7678.6497	80.3884442	83.3865207	6136.21399	**0**	124.098713
Immune dysregulation-polyendocrinopathy-enteropathy-X-linked syndrome	log-normal	-7810.2545	81.0079388	84.9557328	6439.5122	**0**	100.355543
Immunodeficiency 10 - 1	log-normal	-2298.5424	22.1146492	24.715437	919.848215	**0**	37.6270794
Immunodeficiency 11A	log-normal	-1146.8173	19.4126753	25.7500101	827.498486	**0**	27.4030016
Immunodeficiency 13	log-normal	-1502.1959	26.0882017	30.0769675	1006.90028	**0**	45.2048272
Immunodeficiency 14	log-normal	-2994.4395	66.9568318	69.0377726	2261.95051	**0**	77.9938685
Immunodeficiency 16	log-normal	-1648.967	52.0375221	65.6193376	1030.26452	**0**	59.7456949
Immunodeficiency 23	log-normal	-1144.6026	10.5484559	32.287515	769.483856	**0**	30.1589433
Immunodeficiency 35	log-normal	-2322.139	65.3435149	83.5326296	1555.39289	**0**	76.059587
Immunodeficiency 40	log-normal	-1938.2428	19.1008639	37.1145653	1403.56015	**0**	35.9129171
Immunodeficiency 41	log-normal	-1345.1972	35.8016492	42.4817839	841.091095	**0**	41.516375
Immunodeficiency 43	log-normal	-640.02415	8.22924787	11.5032115	346.930341	**0**	15.7236927
Immunodeficiency 73a with defective neutrophil chemotaxis and leukocytosis	log-normal	-782.1895	6.28758532	11.3978519	483.459363	**0**	15.0547838
Immunodeficiency with hyper IgM type 3	log-normal	-2107.4686	44.6221934	63.9664362	1478.72148	**0**	57.7460952
Immunodeficiency with hyper-IgM type 2	log-normal	-2554.4355	61.2683079	72.1785489	1853.54653	**0**	71.7780273

PPI network	Best model	AIC_{min}	ΔAIC_e	ΔAIC_w	ΔAIC_p	ΔAIC_{ln}	ΔAIC_{pc}
Immunodeficiency-centromeric instability-facial anomalies syndrome	log-normal	-4544.0374	2.23438024	28.3571077	3025.91172	**0**	57.2564998
Immunoglobulin alpha deficiency	log-normal	-4768.8874	51.7851837	66.0593442	3786.9311	**0**	94.5844063
Inappropriate ADH syndrome	log-normal	-1466.1661	4.60019583	14.2512713	942.744064	**0**	18.5797593
Intermediate coronary syndrome	log-normal	-7746.457	99.8536777	105.921736	6539.64196	**0**	112.672548
Intermediate spinal muscular atrophy	log-normal	-1181.3885	3.89587692	18.7213018	882.46128	**0**	23.1238395
Intermediate uveitis	log-normal	-1358.8122	32.2549926	34.2583377	924.624686	**0**	42.3220969
Intermittent asthma	log-normal	-1267.3198	8.49585234	12.5866268	913.008059	**0**	27.7620144
Intermittent claudication	log-normal	-3672.9762	50.6118436	53.8176438	2927.85589	**0**	60.3137268
Intermittent explosive disorder	log-normal	-803.52047	4.34808096	6.51851674	601.279667	**0**	18.3242784
Interstitial keratitis	log-normal	-727.92397	10.6531959	13.3327136	535.601062	**0**	19.8103148
Interstitial lung disease	log-normal	-20692.85	41.5619204	57.1436417	18813.281	**0**	84.29907
Interstitial nephritis	log-normal	-5892.4394	56.1191254	59.6702329	4529.66984	**0**	75.034719
Iron deficiency anemia	log-normal	-5653.0137	55.2669771	58.0593921	4749.6671	**0**	77.8121055
Iron metabolism disease	log-normal	-3508.6405	30.866476	33.5013434	2986.6405	**0**	47.6557307
Isolated growth hormone deficiency type IA	log-normal	-6010.805	76.9407207	79.0232303	4714.61001	**0**	100.448636
Juvenile absence epilepsy	log-normal	-1700.1703	4.636888	7.63770417	1089.79188	**0**	30.3756149
Juvenile myelomonocytic leukemia	log-normal	-6908.9703	93.2687718	95.282966	5408.66033	**0**	108.543887
Kidney angiomyolipoma	log-normal	-2238.2014	34.050879	36.8733446	1192.68183	**0**	7.38969239
Kidney rhabdoid cancer	log-normal	-1259.1877	9.49864854	13.6768254	951.365031	**0**	20.0915525
Left bundle branch hemiblock	log-normal	-2620.5566	54.1729577	59.7353402	2039.47422	**0**	66.4662362
Leprosy	log-normal	-786.19663	15.545507	22.0491545	518.460752	**0**	21.774771
Lipoma	log-normal	-6693.838	3.57956927	35.7767243	5706.12853	**0**	77.5364983
Liver disease	log-normal	-20486.376	34.6792628	102.326028	18156.1652	**0**	141.413836
Llethal congenital contracture syndrome 1	log-normal	-1744.6516	36.1304931	39.6568399	1274.67411	**0**	45.8813729
Llupus erythematosus	log-normal	-21041.493	68.6563416	70.7993082	19331.3882	**0**	92.0526285
Llymphocytic choriomeningitis	log-normal	-3251.1431	85.0946305	87.9395789	1947.71862	**0**	92.9687977
Lung abscess	log-normal	-1295.7531	22.1760371	24.2998219	1001.92647	**0**	35.1687582
Lymphatic system disease	log-normal	-21320.674	37.5901541	116.462725	14409.9128	**0**	152.771523
Meckel syndrome	log-normal	-9157.5968	93.9892018	112.511394	7162.8641	**0**	130.037637
Mikulicz disease	log-normal	-1001.6515	20.3276646	24.2088012	643.700629	**0**	26.3529684
Mitochondrial DNA depletion syndrome	log-normal	-6050.5433	77.5506998	80.3252093	4851.4778	**0**	98.9523434
Mitochondrial encephalomyopathy	log-normal	-8014.2103	88.789975	94.0369701	6387.48997	**0**	109.980174
Mitochondrial myopathy	log-normal	-14032.091	81.2730836	155.96836	11576.6025	**0**	167.951851
Mitral valve edge	log-normal	-8537.9023	52.8798294	56.4546528	7421.33641	**0**	79.6373539

**PPI network**	**Best model**	**AIC_{min}**	**ΔAIC_e**	**ΔAIC_w**	**ΔAIC_p**	**ΔAIC_{ln}**	**ΔAIC_{pc}**
Motor peripheral neuropathy	log-normal	-4788.5136	19.0314648	35.2833743	4085.4136	**0**	56.4770235
Multiple benign circumferential skin creases on limbs	log-normal	-707.26051	11.3319919	13.3384369	443.665566	**0**	19.9421105
Multiple carboxylase deficiency	log-normal	-2072.8526	3.16593085	31.2487945	1536.60651	**0**	36.9668869
Multiple chemical sensitivity	log-normal	-919.55287	11.4939549	19.4151109	623.3731	**0**	21.5711948
Multiple congenital anomalies-hypotonia-seizures syndrome	log-normal	-572.25121	**0.1635442**	2.98437533	21.9665944	**0**	10.7386333
Multiple endocrine neoplasia	log-normal	-8246.2597	74.8034359	77.4292428	6811.89672	**0**	89.6389331
Multiple mitochondrial dysfunctions syndrome - 1	log-normal	-1323.503	28.1146367	44.3237967	664.366159	**0**	40.5088898
Multiple mitochondrial dysfunctions syndrome	log-normal	-1323.503	28.1146367	44.3237967	664.366159	**0**	40.5088898
Multiple myeloma	log-normal	-20997.265	36.3784778	117.894522	14904.9958	**0**	150.970791
Multiple pterygium syndrome	log-normal	-880.37173	**0.43618422**	2.83019466	440.029893	**0**	16.4003883
Multiple synostoses syndrome	log-normal	-1029.2286	2.34921154	9.19721977	696.306924	**0**	18.3904799
Multiple system atrophy	log-normal	-7983.846	57.3207485	60.0476075	6528.81891	**0**	77.6465918
Muscle benign neoplasm	log-normal	-5659.9105	31.8861078	43.0442992	4227.96912	**0**	67.0306983
NUT midline carcinoma	log-normal	-4295.9069	84.5370249	88.5372708	3336.87839	**0**	108.248265
Nasu–Hakola disease	log-normal	-3493.4589	36.0065655	38.0074537	2700.35663	**0**	51.3753231
Neonatal abstinence syndrome	log-normal	-752.96089	2.26560293	5.63913216	447.895742	**0**	16.9815208
Nephrosclerosis	log-normal	-3826.9578	37.7954558	43.3099571	3169.22498	**0**	62.4569782
Neuroendocrine carcinoma	log-normal	-5786.3558	51.1681334	62.0133665	4321.60104	**0**	87.3191558
Neuronal ceroid lipofuscinosis 3	log-normal	-6323.6343	41.1009363	56.4117338	5372.50372	**0**	68.7565122
Nijmegen breakage syndrome	log-normal	-6954.2393	167.405578	176.963994	5047.1298	**0**	178.662116
Noonan syndrome with multiple lentigines	log-normal	-4144.5752	67.0886199	71.0643432	3379.72629	**0**	78.8336471
Ocular motility disease	log-normal	-13671.605	55.3998148	122.626915	10559.066	**0**	147.032833
Ovary adenocarcinoma	log-normal	-16875.701	13.2274823	114.647321	13156.3377	**0**	192.167436
Pain agnosia	log-normal	-20134.508	87.9189458	98.4074969	17477.8723	**0**	123.69568
Paranasal sinus disease	log-normal	-10191.791	63.8827993	73.8228805	8956.51182	**0**	96.555543
Pericarditis	log-normal	-6345.0741	82.5130986	84.5407879	5140.17146	**0**	94.5498879
Peritoneum cancer	log-normal	-4262.127	99.4235488	101.809535	3123.84509	**0**	116.370026
Peutz–Jeghers syndrome	log-normal	-6109.6114	54.5149487	58.6695049	4362.30518	**0**	76.7477837
Phosphorus metabolism disease	log-normal	-7383.6077	81.3546584	83.355021	6050.86733	**0**	95.8345652
Pleural disease	log-normal	-15861.641	128.519723	134.739159	13523.8385	**0**	148.662842
Pneumoconiosis	log-normal	-8108.5343	124.233849	126.472591	6928.63073	**0**	139.537742
Polycystic liver disease	log-normal	-3887.5977	44.4868427	51.4844307	2641.46898	**0**	61.9896676
Polycystic ovary syndrome	log-normal	-18467.274	105.637271	108.256387	15089.4713	**0**	129.838752

PPI network	Best model	AIC_{min}	ΔAIC_e	ΔAIC_w	ΔAIC_p	ΔAIC_{ln}	ΔAIC_{pc}
Porphyria	log-normal	-4760.445	40.5017016	42.6766071	3913.99253	**0**	62.5599912
Primary autosomal recessive microcephaly 2 with or without cortical malformations	log-normal	-1102.8911	3.54528337	14.1865685	837.419119	**0**	17.4855188
Primary bacterial infectious disease	log-normal	-21613.333	17.9517481	31.6787893	19962.8339	**0**	61.0712067
Primary biliary cholangitis	log-normal	-16128.91	81.8393888	101.96445	14744.5072	**0**	131.216577
Primary congenital glaucoma default edge	log-normal	-2777.1313	4.52844078	21.6165276	2115.13243	**0**	39.7120384
Primary hyperoxaluria	log-normal	-21435.658	266.073928	268.087032	15869.2708	**0**	292.832554
Primary hyperparathyroidism	log-normal	-2765.0727	35.8656042	38.8358753	2090.51481	**0**	48.2677912
Primary hypoalphalipoproteinemia 1	log-normal	-890.31577	**0.2177886**	4.40247761	632.252201	**0**	23.4443236
Primary immunodeficiency disease	log-normal	-21521.143	8.82256295	16.2736264	17237.5425	**0**	38.3298849
Progressive familial heart block	log-normal	-1440.3862	24.8629333	35.2003288	906.306142	**0**	36.564858
Progressive familial intrahepatic cholestasis	log-normal	-2948.8067	9.19355847	11.2259405	2342.86759	**0**	30.0278928
Progressive multifocal leukoencephalopathy	log-normal	-964.90887	33.7329989	65.4890073	508.529254	**0**	49.1844612
Progressive myoclonus epilepsy	log-normal	-5844.0559	22.3489953	25.2472489	4570.04133	**0**	55.1473574
Progressive relapsing multiple sclerosis	log-normal	-521.18342	7.14396599	11.125377	349.735563	**0**	13.3898437
Progressive supranuclear palsy	log-normal	-9074.0703	74.0383417	76.1323186	7614.97214	**0**	101.315339
Prostate adenocarcinoma	log-normal	-19119.225	124.371453	166.625224	15286.2728	**0**	213.398625
Pulmonary fibrosis	log-normal	-20431.995	67.3656588	74.7676142	18375.5309	**0**	97.6150899
Renal cell carcinoma	log-normal	-19729.144	52.9254655	100.056715	15953.5505	**0**	129.317848
Renal fibrosis	log-normal	-17395.488	99.779351	103.338945	15027.4823	**0**	121.427681
Renal hypertension	log-normal	-5769.5931	50.7194653	52.8078031	4871.90058	**0**	63.6157991
Renal hypoplasia	log-normal	-4164.0921	19.2707565	23.042285	3166.91235	**0**	48.3484794
Renal oncocytoma	log-normal	-3144.3897	9.94473445	13.3401569	2806.09274	**0**	45.6065461
Restrictive cardiomyopathy	log-normal	-4064.1084	72.493049	76.9044803	3056.36918	**0**	86.3694668
Rheumatoid arthritis	log-normal	-21721.549	82.3619025	84.6751673	19941.4245	**0**	105.787165
SAPHO syndrome	log-normal	-1368.1804	8.30153124	11.0172347	1078.95043	**0**	17.3394588
Selective immunoglobulin deficiency disease	log-normal	-2177.7194	28.2803736	31.8272406	1717.6775	**0**	49.0391324
Small cell carcinoma	log-normal	-8796.695	76.6906425	78.7985505	7686.47027	**0**	92.3617739
Small cell osteogenic sarcoma	log-normal	-859.88387	6.60391554	11.1133492	632.808251	**0**	18.1278599
Small intestine benign neoplasm	log-normal	-1325.8367	6.10589159	8.25729666	1087.89164	**0**	21.845885
Small intestine cancer	log-normal	-4577.8031	20.2086407	29.5514128	3416.71597	**0**	51.7579728
Small intestine carcinoma	log-normal	-2069.088	15.3836541	19.4231629	1663.0207	**0**	39.6747332
Smallpox	log-normal	-7913.4848	121.148959	128.393395	6541.18096	**0**	150.309741
Spondyloarthropathy	log-normal	-5945.42	101.122353	105.264835	4674.6817	**0**	122.550052
Stomach cancer	log-normal	-18238.345	123.135874	144.444605	14574.4172	**0**	162.085192

PPI network	Best model	AIC_{min}	ΔAIC_e	ΔAIC_w	ΔAIC_p	ΔAIC_{ln}	ΔAIC_{pc}
Stroke	log-normal	-927.81486	14.1225835	46.9721273	540.702102	**0**	35.5435929
Systemic scleroderma	log-normal	-20580.196	130.653014	136.937756	18585.8471	**0**	160.724812
Testicular disease	log-normal	-19214.449	72.1082037	119.923217	16109.6745	**0**	170.172585
Tetanus	log-normal	-18323.458	74.1431556	84.4392016	16887.4259	**0**	106.070127
Third-degree atrioventricular block	log-normal	-1827.0395	17.7663876	20.010219	1458.21033	**0**	35.021672
Thyroid dyshormonogenesis	log-normal	-2509.3561	8.32876134	18.455397	1997.68967	**0**	31.8653569
Thyroid gland anaplastic carcinoma	log-normal	-6353.5373	39.8152061	72.4948036	5443.87334	**0**	115.836671
Thyroid gland cancer	log-normal	-13675.8	103.475698	138.775694	10350.8415	**0**	180.440774
Thyroid gland carcinoma	log-normal	-13154.755	89.7855363	127.419965	9810.98477	**0**	26.913553
Thyroid gland disease	log-normal	-20817.568	117.109458	124.222427	16945.4689	**0**	146.81044
Thyroid gland medullary carcinoma	log-normal	-6440.6632	64.2270828	72.7676784	5211.40374	**0**	94.523895
Thyroid hormone resistance syndrome	log-normal	-1215.209	2.48723564	6.70454081	991.384562	**0**	18.5125603
Thyroiditis	log-normal	-7126.8659	119.563633	122.008594	5481.51808	**0**	135.575503
Urea cycle disorder	log-normal	-5808.8189	38.7157693	41.7391444	4663.96042	**0**	60.8556261
Urticaria	log-normal	-9309.3143	57.1209538	62.2897079	7165.02153	**0**	79.0205992
Vascular cancer	log-normal	-10749.073	139.264379	141.431116	8911.78396	**0**	155.577316
Ventricular septal defect	log-normal	-16625.361	75.4741639	86.2873931	13741.4129	**0**	125.601594
Werdnig–Hoffmann disease	log-normal	-2224.9475	21.1381167	27.4310833	1682.3893	**0**	40.5294161
X-linked agammaglobulinemia	log-normal	-6563.9956	96.2761551	99.8955634	4988.1585	**0**	116.463264
ankylosing spondylitis	log-normal	-17934.58	116.499129	138.819244	14827.5508	**0**	169.89043
Autosomal recessive spinocerebellar ataxia 8	log-normal	-1513.6092	**1.97343714**	11.2576283	955.28793	**0**	28.5713417
Bone giant cell tumor	log-normal	-4344.4025	58.9928817	61.5552187	2934.96922	**0**	75.720541
Bone osteosarcoma	log-normal	-8961.2874	62.0185581	71.4283924	6801.87603	**0**	97.7061815
Chronic recurrent multifocal osteomyelitis	log-normal	-3285.5054	47.4580622	50.2416086	2574.32819	**0**	63.6523472
Cleidocranial dysplasia	log-normal	-4800.6619	11.6270808	19.1398314	3702.48026	**0**	43.382951
Exostosis	log-normal	-5375.0787	43.8009457	52.7864766	4535.665	**0**	82.8356981
Hepatitis A	log-normal	-8069.0738	107.1122	109.112368	6930.28598	**0**	120.496552
Hepatitis E	log-normal	-4006.0188	15.9959549	31.4187802	3244.86669	**0**	62.7340397
Immunodeficiency 21	log-normal	-5542.1475	75.3892079	82.5657361	4527.70783	**0**	105.882731
Megaloblastic anemia	log-normal	-3497.1635	26.0442954	32.2089251	2757.26332	**0**	46.6804986
Mucopolysaccharidosis IV	log-normal	-2452.4722	8.45446272	14.2526068	1870.14574	**0**	35.4472136
Osteoarthritis	log-normal	-19791.541	130.760412	134.806292	17969.2031	**0**	154.699724
Osteoblastoma	log-normal	-2399.8287	15.4262889	18.2834719	1595.30617	**0**	33.8858566
Osteochondritis dissecans	log-normal	-2300.0432	9.79557566	15.14133	1715.00988	**0**	37.5670165

PPI network	Best model	AIC_{min}	ΔAIC_e	ΔAIC_w	ΔAIC_p	ΔAIC_{ln}	ΔAIC_{pc}
Osteogenesis imperfecta type 1	log-normal	-1209.2993	5.28308928	11.6762359	864.235537	**0**	22.5484174
Osteogenesis imperfecta type 12	log-normal	-626.85335	3.27734109	7.14685069	378.202699	**0**	14.2051514
Osteogenesis imperfecta type 15	log-normal	-702.99077	3.01037359	5.60946478	475.725487	**0**	17.2622853
Osteogenesis imperfecta type 6	log-normal	-522.49295	**0.7602711**	6.50407792	277.815193	**0**	9.39871592
Osteoglophonic dysplasia	log-normal	-1250.8122	5.19079112	8.58387882	954.474085	**0**	17.2120263
Osteomalacia	log-normal	-6142.7516	37.2387497	39.8298491	5426.44169	**0**	55.6129599
Osteomyelitis	log-normal	-12135.334	52.3200875	78.9844347	10115.4915	**0**	110.169156
Osteonecrosis	log-normal	-12595.436	171.61494	175.880853	10618.5454	**0**	195.937103
Osteosarcoma	log-normal	-17335.15	246.121103	264.113583	13019.644	**0**	274.414076
Osteosclerosis	log-normal	-17657.41	74.5656146	87.8251974	15339.2625	**0**	114.368101
Recessive dystrophic epidermolysis bullosa	log-normal	-3525.666	9.72232299	21.9123554	2833.94745	**0**	50.580232
Syndactyly type 3	log-normal	-1141.8001	**1.13610573**	34.4436928	791.04019	**0**	29.9507582
Syndactyly-telecanthus-anogenital and renal malformations syndrome	log-normal	-1237.7644	8.9069195	11.5249722	936.015266	**0**	25.0563685
Syndactyly	log-normal	-15495.084	15.9030347	18.1652636	12980.8159	**0**	62.4586847
Syndromic microphthalmia 9	log-normal	-1216.3312	3.92103578	8.16491268	653.912584	**0**	21.3908148
Tibial muscular dystrophy	log-normal	-2880.4803	24.5872637	27.4351422	2352.69945	**0**	49.1951089
Transthyretin amyloidosis	log-normal	-4921.986	61.8609263	63.9849375	4274.67554	**0**	78.0121953
Viral encephalitis	log-normal	-8517.0289	131.952548	140.180468	7118.46552	**0**	145.388667
Viral exanthem	log-normal	-1404.63	7.70438339	9.79570492	1070.99541	**0**	20.7249593
Viral hepatitis	log-normal	-20100.243	10.1738419	62.3030998	18306.6624	**0**	101.471207
Viral meningitis	log-normal	-3323.1201	71.1640093	73.2122901	2524.22186	**0**	83.5553568
Viral pneumonia	log-normal	-8172.5814	144.462149	147.300768	5985.91293	**0**	155.659357
Choroideremia	power law with cutoff	-3532.6123	23.784517	26.4932072	2148.37766	13.834404	**0**
Hereditary spastic paraplegia 35	power law with cutoff	-1914.6964	12.0034849	18.1097751	1037.21034	35.9187599	**0**
Multiple acyl-CoA dehydrogenase deficiency	power law with cutoff	-3090.8208	52.2928763	61.0675796	1811.6408	32.7522963	**0**
Syndromic intellectual disability	power law with cutoff	-9124.3139	75.1610584	77.1698141	5393.84969	92.8033482	**0**
Autophagy	exponential	-58552.9635	**0**	5892.45381	41694.87937	12987.94970	14621.28902
Glioblastoma	exponential	-58536.5835	**0**	6092.21364	39778.71763	14612.34616	15940.28399

Table S3 shows the model selection of fractal Equation (6), exponential Equation (7), delayed fractal Equation (8), and delayed exponential Equation (9) functions of box covering of the networks based on AIC. The ΔAIC_f, ΔAIC_e, ΔAIC_{df}, ΔAIC_{de}, correspond to fractal, exponential, delayed fractal, and delayed exponential, respectively. The AIC of each model were not reported in Table S2 for simplicity, but $AIC_i = \Delta AIC_i + AICmin$. The best model is based on ΔAIC=0.

Table S3. The ΔAIC of 476 human PPI networks for box covering, the values in bold are those two less than two.

PPI network	Nodes	Best model	AIC_{min}	ΔAIC_f	ΔAIC_e	ΔAIC_{df}	ΔAIC_{de}
Primary hyperparathyroidism	308	delayed exponetial	22.9759537	34.2943493	**0.165385468**	67.8899912	**0**
Charcot–Marie–Tooth disease type 2B1	243	delayed exponetial	29.2304806	35.5735394	**0.213893212**	69.31590348	**0**
Renal adenoma	142	delayed exponetial	13.3366381	35.8780219	**0.223671035**	66.61246148	**0**
Syndromic intellectual disability	1070	delayed exponetial	58.0512718	46.9812209	**0.242315011**	91.81790881	**0**
Nose disease	1922	delayed exponetial	56.5507888	36.2438902	**0.285313271**	75.14997702	**0**
Cervix carcinoma	1594	delayed exponetial	53.2677619	37.3899584	**0.361069689**	75.4547566	**0**
Ocular motility disease	1452	delayed exponetial	46.3464254	41.5448301	**0.394900541**	80.85556668	**0**
Cleft lip-palate-ectodermal dysplasia syndrome	92	delayed exponetial	15.4429902	36.0382227	**0.401955731**	67.47523042	**0**
Colorectal adenocarcinoma	1723	delayed exponetial	58.4795978	40.7463989	**0.50180218**	86.18741167	**0**
Breast secretory carcinoma	93	delayed exponetial	19.8549082	18.2833852	**0.686785288**	2.973316381	**0**
Autosomal dominant non-syndromic intellectual disability	1034	delayed exponetial	53.7179148	40.6264904	**0.757616191**	81.79172079	**0**
Neuronal ceroid lipofuscinosis 3	691	delayed exponetial	35.3013499	41.1258521	**0.990759641**	80.01025387	**0**
Coloboma	1342	delayed exponetial	50.2652041	46.3282628	**1.500220154**	89.89763977	**0**
Mitral valve edge	867	delayed exponetial	30.1188336	50.164215	**1.545550692**	88.83829771	**0**
Nephrosclerosis	399	delayed exponetial	45.5968346	27.7391899	**1.557126397**	72.6476966	**0**
Prostate adenocarcinoma	1765	delayed exponetial	52.1150872	39.0801068	**1.595359472**	78.21944662	**0**
Progressive bulbar palsy	194	delayed exponetial	34.4924539	26.6555379	**1.651625107**	60.37771045	**0**
Colonic benign neoplasm	1130	delayed exponetial	58.4671413	34.3279405	**1.687067116**	78.61283915	**0**
Charcot–Marie–Tooth disease type 1A	601	delayed exponetial	40.9470311	36.0555413	**1.742170463**	72.07860907	**0**
Charcot–Marie–Tooth disease type 4D	206	delayed exponetial	42.5462209	30.8175757	**1.805099068**	64.17130321	**0**
Primary cutaneous amyloidosis	342	delayed exponetial	38.5201203	33.3348042	**1.844640407**	65.58588059	**0**
Amyotrophic lateral sclerosis type 2	345	delayed exponetial	57.9200948	27.4303534	**1.877705127**	69.29384615	**0**
Disorder of sexual development	1888	delayed exponetial	57.2098525	45.6831853	2.013827405	89.13899516	**0**
Interstitial nephritis	575	delayed exponetial	36.8623836	32.9217392	2.141700172	75.45132235	**0**
Hyper IgM syndrome	597	delayed exponetial	24.3716214	36.1871413	2.163683185	75.86426096	**0**
Ankylosing spondylitis	1641	delayed exponetial	60.327567	38.6167197	2.183926213	83.47232896	**0**
Noonan syndrome with multiple lentigines	426	delayed exponetial	29.7837463	36.9944614	2.383329552	77.73990067	**0**
Meckel's diverticulum	263	delayed exponetial	34.8955616	29.1590257	2.650898132	64.81910333	**0**
Small intestine carcinoma	243	delayed exponetial	30.6922615	37.9961247	2.660644593	78.68826614	**0**

PPI network	Nodes	Best model	AIC_{min}	ΔAIC_f	ΔAIC_e	ΔAIC_{df}	ΔAIC_{de}
Charcot–Marie–Tooth disease type 4C	290	delayed exponetial	38.0287917	36.9564618	2.695711935	74.69829504	**0**
Testicular disease	1813	delayed exponetial	51.9813648	40.4011753	2.716244607	78.79607528	**0**
Vascular cancer	982	delayed exponetial	38.0013074	33.7880117	2.762859448	69.22870364	**0**
Cocaine abuse	509	delayed exponetial	41.9001072	28.5053726	2.788368706	68.44034616	**0**
Thyroid gland medullary carcinoma	624	delayed exponetial	51.5003006	39.7860624	2.807845816	44.58787813	**0**
Osteomyelitis	1133	delayed exponetial	41.6393548	42.4007131	2.934006736	81.60937728	**0**
Thyroiditis	647	delayed exponetial	32.5148759	33.0408126	3.10657919	68.8835512	**0**
Colon adenocarcinoma	1670	delayed exponetial	56.2260247	41.2651909	3.139925031	87.86916546	**0**
Syndromic X-linked intellectual disability	830	delayed exponetial	45.4443911	46.4808387	3.167223484	86.14598831	**0**
Immunodeficiency 21	541	delayed exponetial	33.035264	29.9194077	3.169172722	65.73835902	**0**
Carney–Stratakis syndrome	118	delayed exponetial	2.29108874	38.2418522	3.177338988	18.86261753	**0**
Gilles de la Tourette syndrome	834	delayed exponetial	51.5768716	40.8229241	3.202058242	80.12933033	**0**
Iron metabolism disease	399	delayed exponetial	25.1713289	43.4914608	3.343690619	81.30444019	**0**
Viral pneumonia	741	delayed exponetial	40.7347127	27.0064817	3.357216757	62.57561422	**0**
Meckel syndrome	1005	delayed exponetial	33.406882	49.8873305	3.370773956	87.90244024	**0**
Charcot–Marie–Tooth disease type 4B2	304	delayed exponetial	28.3754615	32.1081355	3.382635489	62.36225895	**0**
Cleidocranial dysplasia	515	delayed exponetial	35.8574923	36.2110469	3.461793975	74.69932384	**0**
Congenital adrenal hyperplasia	427	delayed exponetial	13.0678448	49.2225982	3.78030705	82.39905321	**0**
Muscle benign neoplasm	572	delayed exponetial	37.2224255	37.7893045	3.793979862	75.10140573	**0**
Selective immunoglobulin deficiency disease	242	delayed exponetial	36.4513541	22.1925532	3.794375796	61.90038364	**0**
Osteonecrosis	1143	delayed exponetial	49.4280606	34.6108535	3.932189621	73.96562755	**0**
Brachydactyly type B2	102	delayed exponetial	1.47384012	38.7027912	3.938602735	19.92191253	**0**
Charcot–Marie–Tooth disease type 1E	220	delayed exponetial	22.3366401	32.0935445	3.997617281	63.71488117	**0**
Osteosclerosis	1631	delayed exponetial	44.7064323	34.7490505	4.232888232	69.6317604	**0**
Charcot–Marie–Tooth disease type 4F	199	delayed exponetial	33.5746903	39.7887289	4.479215777	82.64037674	**0**
Hyperprolactinemia	393	delayed exponetial	27.9485549	29.595502	4.500163089	66.33225603	**0**
Exostosis	564	delayed exponetial	37.7484149	35.6205526	4.625526489	74.24902008	**0**
Urticaria	876	delayed exponetial	38.9443358	31.9175822	4.836124845	66.70052074	**0**
Llymphocytic choriomeningitis	305	delayed exponetial	26.047669	19.4554357	4.865165328	53.14033292	**0**
Hepatitis A	743	delayed exponetial	34.5715457	32.6672947	4.968773677	68.77811247	**0**
Charcot–Marie–Tooth disease type 1F	221	delayed exponetial	46.5342842	24.3845628	5.114137313	71.60804326	**0**
Immunodeficiency-centromeric instability-facial anomalies syndrome	485	delayed exponetial	49.7712897	28.7830148	5.266679803	71.9839173	**0**
Small cell osteogenic sarcoma	120	delayed exponetial	20.3721318	25.893144	5.286455025	57.1256933	**0**
Immunodeficiency 41	139	delayed exponetial	26.5917922	8.8486804	5.370882359	43.04840647	**0**

PPI network	Nodes	Best model	AIC_{min}	ΔAIC_f	ΔAIC_e	ΔAIC_{df}	ΔAIC_{de}
Chronic obstructive pulmonary disease	1862	delayed exponetial	50.7669378	39.8159979	5.382022414	80.43039097	**0**
Melnick–Needles syndrome	238	delayed exponetial	58.357136	28.1538438	5.38873323	72.12600438	**0**
Charcot–Marie–Tooth disease type 4G	119	delayed exponetial	18.7893523	33.5564392	5.450911363	5.081616132	**0**
Lattice corneal dystrophy	117	delayed exponetial	32.7086128	19.7529183	5.510726598	53.86446898	**0**
NUT midline carcinoma	421	delayed exponetial	28.7870038	39.2710718	5.517548333	78.53722187	**0**
Drug dependence	824	delayed exponetial	63.3712092	34.3334144	5.547610986	81.16216941	**0**
Paranasal sinus disease	967	delayed exponetial	43.2298835	37.9083823	5.548919881	77.49366051	**0**
Spondyloarthropathy	559	delayed exponetial	39.7134627	20.1847447	5.71778456	59.47329935	**0**
Bone osteosarcoma	880	delayed exponetial	20.109804	59.5617698	5.796080305	99.06320155	**0**
Cardiac arrest	1385	delayed exponetial	44.0764586	42.1222349	6.430884882	82.38550564	**0**
Arthritis	1952	delayed exponetial	54.9525194	36.2024394	6.435665497	77.00844184	**0**
Immune dysregulation-polyendocrinopathy-enteropathy-X-linked syndrome	725	delayed exponetial	45.795566	31.7937863	6.672011028	70.33389431	**0**
Brachydactyly type A1	124	delayed exponetial	27.8342671	3.57343541	7.050879853	**0.459291988**	**0**
Factor VIII deficiency	219	delayed exponetial	17.8426834	32.8704572	7.116715604	68.22758188	**0**
Selective IgA deficiency disease	158	delayed exponetial	46.5407715	19.7045823	7.393882918	64.79065817	**0**
Neuroendocrine carcinoma	577	delayed exponetial	39.1166505	37.3124426	7.396540481	73.36937915	**0**
Cardiovascular cancer	1018	delayed exponetial	34.9588092	37.610202	7.723935035	72.7928401	**0**
Autosomal dominant cerebellar ataxia, deafness and narcolepsy	164	delayed exponetial	40.2718663	18.5616972	7.797295729	61.78492201	**0**
Osteopathia striata with cranial sclerosis	135	delayed exponetial	36.0599968	29.0521901	7.832490882	72.27142755	**0**
Alpers–Huttenlocher syndrome	412	delayed exponetial	29.1353907	29.7876748	7.877177728	65.77401217	**0**
Charcot–Marie–Tooth disease type 4H	260	delayed exponetial	31.4023901	31.9885263	7.88774252	68.11977071	**0**
Syndactyly-telecanthus-anogenital and renal malformations syndrome	169	delayed exponetial	35.5075481	17.4599836	8.009336482	56.93204993	**0**
Charcot–Marie–Tooth disease type 4B1	328	delayed exponetial	21.3224195	44.7756899	8.385334215	81.99481923	**0**
Progressive multifocal leukoencephalopathy	96	delayed exponetial	6.3852877	17.7162208	8.386380954	2.377605598	**0**
Viral encephalitis	776	delayed exponetial	19.8006258	45.5659438	8.497982497	84.12375968	**0**
Mitochondrial DNA depletion syndrome	648	delayed exponetial	50.8392179	28.76136	8.519496167	76.09077806	**0**
Hemophilia B	306	delayed exponetial	28.3226381	36.7684291	8.571177155	73.87342616	**0**
Hypothyroidism	1929	delayed exponetial	48.3566212	43.4816027	8.656879493	83.41432924	**0**
Intermittent claudication	371	delayed exponetial	16.6609887	40.0096011	8.738484704	76.8593814	**0**
Hyperuricemia	803	delayed exponetial	46.3724677	37.8684785	9.044063555	84.47462104	**0**
Childhood acute myeloid leukemia	461	delayed exponetial	32.0753859	51.8226336	9.069820494	89.02503565	**0**
Osteosarcoma	1527	delayed exponetial	45.6538941	31.5197379	9.194650365	67.80095786	**0**
Cervix uteri carcinoma in situ	335	delayed exponetial	27.9931338	46.1090997	9.257161885	87.17933078	**0**
Camurati–Engelmann disease	194	delayed exponetial	30.0320304	25.6520748	9.289206935	64.75394813	**0**

PPI network	Nodes	Best model	AIC_{min}	ΔAIC_f	ΔAIC_e	ΔAIC_{df}	ΔAIC_{de}
Mucopolysaccharidosis IV	336	delayed exponetial	45.9029827	28.2829631	9.395671206	69.23098708	**0**
Cholesterol embolism	115	delayed exponetial	12.2959428	23.7595279	9.474935864	8.042412364	**0**
Crohn colitis	260	delayed exponetial	33.3979533	41.7495495	9.564710794	88.07626224	**0**
Drug psychosis	175	delayed exponetial	35.9789108	33.151639	9.600848716	77.52560143	**0**
Autosomal dominant Emery-Dreifuss muscular dystrophy 2	268	delayed exponetial	18.4666794	40.684539	9.687228057	70.51847732	**0**
Cecal disease	471	delayed exponetial	38.3918866	36.182318	9.801948839	82.82925092	**0**
Aortic disease	1745	delayed exponetial	48.9977078	41.5114716	9.832453581	81.17964531	**0**
Charcot–Marie–Tooth disease type X	377	delayed exponetial	30.4276567	38.3314656	10.26871588	75.07743701	**0**
Multiple mitochondrial dysfunctions syndrome - 1	153	delayed exponetial	25.7226541	15.2392271	10.36713105	55.2006148	**0**
Multiple mitochondrial dysfunctions syndrome	153	delayed exponetial	25.7226541	15.2392271	10.36713105	55.2006148	**0**
Intermediate coronary syndrome	718	delayed exponetial	30.9609664	36.3877447	10.37405242	71.92449499	**0**
Cervical cancer	1637	delayed exponetial	51.8136824	35.6908998	10.40859799	77.34492526	**0**
Drug-induced mental disorder	178	delayed exponetial	37.3270897	32.1017856	10.43498187	76.50462429	**0**
Osteomalacia	622	delayed exponetial	23.0834176	44.1161507	10.54319421	77.73800014	**0**
Burkitt lymphoma	1775	delayed exponetial	43.1875559	35.5898785	10.61702864	72.36693097	**0**
Neuroacanthocytosis	325	delayed exponetial	38.6479018	35.4522434	10.63791319	75.92944567	**0**
Glycogen storage disease VI	170	delayed exponetial	32.2603673	12.9761324	10.82125281	49.96326511	**0**
Progressive supranuclear palsy	944	delayed exponetial	41.9597759	42.0872561	10.86009024	78.43082752	**0**
Primary angle-closure glaucoma	399	delayed exponetial	31.8607632	38.7353286	10.92046227	74.5618861	**0**
Progressive familial heart block	176	delayed exponetial	36.8440044	8.20647581	10.98947102	56.15932752	**0**
Autosomal recessive osteopetrosis 3	148	delayed exponetial	38.5101903	26.5590011	11.28277701	71.53438336	**0**
Systemic scleroderma	1870	delayed exponetial	49.10492	42.4429825	11.3048347	82.18434622	**0**
Hereditary multiple exostoses	192	delayed exponetial	31.4851784	23.83165	11.55107924	63.03447669	**0**
Osteochondrodysplasia	1948	delayed exponetial	38.7644424	43.0566713	11.67274674	78.06630745	**0**
Osteoarthritis	1780	delayed exponetial	42.1975433	37.4996731	11.79054323	73.39563088	**0**
Primary amebic meningoencephalitis	129	delayed exponetial	23.388082	25.8918632	11.82155432	64.78422605	**0**
IgA glomerulonephritis	984	delayed exponetial	41.7539084	42.044284	11.90444134	79.27471733	**0**
Osteogenesis imperfecta type 6	75	delayed exponetial	24.7279536	**1.77176101**	12.01084564	45.75876571	**0**
Lethal congenital contracture syndrome 1	198	delayed exponetial	21.963731	19.1397666	12.01540288	51.83763281	**0**
Leprosy	91	delayed exponetial	13.4995454	14.2557208	12.07391222	**1.391912159**	**0**
Immunodeficiency 13	161	delayed exponetial	34.2795492	8.02530736	12.18503427	57.31709215	**0**
Charcot–Marie–Tooth disease X-linked dominant 1	229	delayed exponetial	22.3152683	38.4975504	12.27034305	75.17399089	**0**
Charcot–Marie–Tooth disease type 2B	515	delayed exponetial	25.1363471	40.7474487	12.27895855	73.05970612	**0**
Uterus carcinoma in situ	335	delayed exponetial	26.5122448	47.2311326	12.42787067	88.64772776	**0**

PPI network	Nodes	Best model	AIC$_{min}$	ΔAIC$_{f}$	ΔAIC$_{e}$	ΔAIC$_{df}$	ΔAIC$_{de}$
Osteogenesis imperfecta type 5	103	delayed exponetial	27.303928	18.8570655	12.46138361	4.114334381	**0**
Chromosome 2q37 deletion syndrome	267	delayed exponetial	38.257961	33.3525421	12.52284691	72.74609469	**0**
Arts syndrome	149	delayed exponetial	33.7382964	23.5248986	12.55470375	66.59282468	**0**
Aicrocephalic osteodysplastic primordial dwarfism type I	297	delayed exponetial	50.303125	24.6844338	12.63599775	73.63917942	**0**
Charcot–Marie–Tooth disease type 2	1003	delayed exponetial	31.398875	61.4046041	12.9207788	103.5871592	**0**
Autosomal recessive spinocerebellar ataxia 8	231	delayed exponetial	46.8340301	25.9965779	12.94990131	72.1141918	**0**
Paranoid schizophrenia	327	delayed exponetial	34.4406742	39.1239263	13.05021546	80.22585142	**0**
Synpolydactyly	359	delayed exponetial	36.4921032	41.5933078	13.14025516	79.90811994	**0**
Polycystic ovary syndrome	1708	delayed exponetial	37.557645	51.5872605	13.148955	92.26515135	**0**
Megaloblastic anemia	420	delayed exponetial	34.2727896	42.1970129	13.41113517	84.91379424	**0**
Lung abscess	150	delayed exponetial	21.1371538	24.2828823	13.74043283	59.44676164	**0**
Renal fibrosis	1576	delayed exponetial	34.4357483	34.4028083	13.84897132	64.63081391	**0**
Isolated growth hormone deficiency type IA	645	delayed exponetial	40.8998436	30.4177085	13.85442765	73.18332177	**0**
Autosomal dominant centronuclear myopathy	305	delayed exponetial	37.5172961	33.4074773	13.89816595	75.8305087	**0**
Renal oncocytoma	392	delayed exponetial	42.5241948	31.7871715	14.00325451	75.32359469	**0**
Syndrome	1972	delayed exponetial	29.0825283	40.3865238	14.05274312	72.69874751	**0**
Urea cycle disorder	643	delayed exponetial	26.195674	57.6445985	14.20731665	100.7254548	**0**
Iimmunodeficiency 60	87	delayed exponetial	23.4793795	19.7480208	14.37759158	7.064828983	**0**
Pleural disease	1413	delayed exponetial	36.8613687	39.4812434	14.72149765	75.50827063	**0**
Segmental dystonia	174	delayed exponetial	30.7685936	29.3491758	14.76877241	72.40337169	**0**
Primary hypoalphalipoproteinemia 1	117	delayed exponetial	19.6490459	29.9132638	14.9753744	66.96520331	**0**
Charcot–Marie–Tooth disease axonal type 2L	244	delayed exponetial	23.8871934	38.4754835	15.04974943	74.61806443	**0**
Primary congenital glaucoma default edge	391	delayed exponetial	42.2922848	33.1202461	15.07006488	75.52724701	**0**
Hereditary spastic paraplegia 35	270	delayed exponetial	24.3418479	39.9469778	15.09077634	75.76946845	**0**
Rheumatoid arthritis	1948	delayed exponetial	54.2098056	38.207634	15.10717407	77.76571928	**0**
Immunodeficiency 10 - 1	276	delayed exponetial	26.3824816	34.1122347	15.17799405	74.07275965	**0**
Primary hypomagnesemia	189	delayed exponetial	35.8527889	25.04977	15.29837013	68.76650979	**0**
Adenoma 2000	1796	delayed exponetial	40.2938211	39.8318447	15.35849891	75.43213472	**0**
Asthma	1924	delayed exponetial	41.842657	39.2458533	15.38358952	74.86739188	**0**
Primary hyperoxaluria	1929	delayed exponetial	37.2217304	44.48772	15.40510687	79.48609717	**0**
Multiple epiphyseal dysplasia	325	delayed exponetial	42.1440525	39.6947685	15.43133585	83.81719948	**0**
Autosomal dominant cerebellar ataxia	1924	delayed exponetial	37.0380465	43.4653554	15.45725812	79.66916944	**0**
Primary hyperaldosteronism	917	delayed exponetial	25.5382816	44.775796	15.57489766	80.77373626	**0**
Autosomal dominant osteopetrosis 2	160	delayed exponetial	29.4168857	34.3847779	15.65073775	72.31434528	**0**

PPI network	Nodes	Best model	AIC_{min}	ΔAIC_f	ΔAIC_e	ΔAIC_{df}	ΔAIC_{de}
Esotropia - 1	546	delayed exponetial	51.7076461	38.3302169	15.8466868	84.56487316	**0**
Polycystic kidney disease	1872	delayed exponetial	36.8159943	53.4343428	15.88811332	94.47568536	**0**
Phosphorus metabolism disease	707	delayed exponetial	18.3444993	49.6580777	16.06244324	84.29756794	**0**
Dyscalculia	231	delayed exponetial	41.3951554	25.1285323	16.33567733	66.77350799	**0**
DNA ligase IV deficiency	196	delayed exponetial	20.9399109	23.5702163	16.35946307	63.48539435	**0**
Thyroid gland disease	1889	delayed exponetial	45.0642169	46.053622	16.44503518	86.38992423	**0**
Amelogenesis imperfecta type 1G	1729	delayed exponetial	40.1583915	39.5411737	16.60073104	75.04686693	**0**
Neuropathy	1978	delayed exponetial	28.3190177	41.054118	16.62197025	73.51352442	**0**
Colorectal carcinoma	1785	delayed exponetial	38.0778445	31.8480468	16.82008651	62.55708612	**0**
Charcot–Marie–Tooth disease recessive intermediate B	116	delayed exponetial	24.9942031	14.9451365	16.94476573	3.876562789	**0**
Colorectal cancer	1765	delayed exponetial	42.7805301	36.7069091	16.96745059	72.72880162	**0**
Chronic recurrent multifocal osteomyelitis	336	delayed exponetial	18.3085763	36.6547033	16.97966676	73.75226372	**0**
Secondary Parkinson disease	220	delayed exponetial	34.0995016	30.3586884	17.00187129	73.29725294	**0**
Dental pulp disease	321	delayed exponetial	41.3411575	21.0155806	17.01007211	72.83814164	**0**
Bile duct disease	1798	delayed exponetial	38.3354365	41.7683517	17.03224213	77.40851495	**0**
Color blindness	527	delayed exponetial	56.5796289	40.0291986	17.04127877	91.19877413	**0**
Amyotrophic lateral sclerosis	1941	delayed exponetial	34.6746504	35.9814298	17.04805069	66.94810216	**0**
Osteogenesis imperfecta type 1	174	delayed exponetial	42.5682931	6.5817956	17.10778581	60.23640867	**0**
Charcot–Marie–Tooth disease axonal type 2S	141	delayed exponetial	28.3103092	26.9883044	17.1371651	70.99406304	**0**
Interstitial lung disease	1848	delayed exponetial	32.5699135	37.2199257	17.4666728	68.44358721	**0**
Glycogen storage disease V	370	delayed exponetial	38.3277711	30.4474697	17.58831092	78.44228321	**0**
Viral meningitis	321	delayed exponetial	23.7241119	26.6727787	17.76830412	67.67101209	**0**
Kidney rhabdoid cancer	159	delayed exponetial	18.3041481	26.3961849	17.77814712	63.07706783	**0**
Renpenning syndrome	143	delayed exponetial	27.4385309	31.6101415	17.92086587	72.16383662	**0**
Multiple myeloma	1832	delayed exponetial	35.3770284	34.0950614	18.1638755	65.57325936	**0**
Nephronophthisis 4	202	delayed exponetial	44.1352791	24.6324269	18.19997874	72.00392123	**0**
Motor neuron disease	1945	delayed exponetial	32.6034436	37.6533774	18.20516648	69.04602913	**0**
Nijmegen breakage syndrome	642	delayed exponetial	23.3792046	38.6270851	18.34679906	77.83573357	**0**
Immunodeficiency 14	298	delayed exponetial	22.5153422	29.1107175	18.38044343	67.82055333	**0**
GRACILE syndrome	267	delayed exponetial	27.8417279	25.9245608	18.40394607	72.04820911	**0**
A amyloidosis	133	delayed exponetial	21.0402856	21.4048396	18.51338877	4.134586946	**0**
Colon carcinoma	1820	delayed exponetial	42.9951363	36.5486989	18.52789434	72.96865178	**0**
Charcot–Marie–Tooth disease type 2A2B	112	delayed exponetial	19.5794463	31.3804254	18.58431977	5.978741422	**0**
Immunoglobulin alpha deficiency	468	delayed exponetial	33.5625652	40.7588156	18.6220777	87.53111466	**0**

PPI network	Nodes	Best model	AIC_{min}	ΔAIC_f	ΔAIC_e	ΔAIC_{df}	ΔAIC_{de}
Bone giant cell tumor	437	delayed exponetial	3.04077676	58.1844978	18.8790973	92.77194953	**0**
Progressive myoclonus epilepsy	697	delayed exponetial	30.1205423	47.1794094	19.13833415	85.23901788	**0**
Werdnig–Hoffmann disease	288	delayed exponetial	38.8457227	34.6343823	19.15614756	84.50519154	**0**
Osteochondrosis	461	delayed exponetial	43.4772462	36.4819192	19.25020645	77.30428489	**0**
Intermediate uveitis	146	delayed exponetial	23.4257568	20.922163	19.57968805	66.65559994	**0**
Gastrointestinal tuberculosis	124	delayed exponetial	16.1686427	29.517674	19.6096783	11.03444552	**0**
Congenital contractural arachnodactyly	283	delayed exponetial	44.9242877	32.3563996	19.71905444	78.05226323	**0**
Glycogen storage disease II	514	delayed exponetial	27.2256655	33.0161802	19.85606144	70.75211883	**0**
Charcot–Marie–Tooth disease type 2E	250	delayed exponetial	34.2761958	46.4853498	19.90930383	97.1060538	**0**
Mitochondrial encephalomyopathy	830	delayed exponetial	36.8255285	46.1867091	19.97247508	94.59503339	**0**
Autosomal dominant distal hereditary motor neuronopathy	477	delayed exponetial	33.0728508	45.9829265	19.98439052	88.40790105	**0**
Autosomal dominant familial periodic fever	412	delayed exponetial	32.1136081	36.252412	19.99600328	86.63424764	**0**
Phosphoglycerate kinase 1 deficiency	161	delayed exponetial	28.350114	31.1243335	20.16003178	73.39116732	**0**
Huntington's disease	1896	delayed exponetial	31.6459747	38.381513	20.4405791	69.71439777	**0**
Renal cell carcinoma	1738	delayed exponetial	41.6326523	38.2040785	20.59812514	73.69062465	**0**
Immunodeficiency with hyper-IgM type 2	270	delayed exponetial	12.183576	31.3808365	20.82168118	65.47465406	**0**
CHIME syndrome	159	delayed exponetial	24.7295336	24.6468376	21.07085531	66.66504088	**0**
Angiodysplasia	135	delayed exponetial	22.9875418	24.534004	21.1966119	65.74460963	**0**
Degenerative disc disease	646	delayed exponetial	17.5420182	48.806799	21.28817681	83.6937031	**0**
Small intestine adenocarcinoma	173	delayed exponetial	24.481649	45.3567139	21.38064383	88.81886301	**0**
Donnai–Barrow syndrome	306	delayed exponetial	49.1850196	39.3725862	21.65071637	86.69580627	**0**
Multiple synostoses syndrome	151	delayed exponetial	26.4285337	36.7474375	21.69502622	84.00523122	**0**
Sarcoma	1895	delayed exponetial	30.9423881	38.1108001	21.73447817	70.42888915	**0**
Inappropriate ADH syndrome	184	delayed exponetial	5.87772668	42.8045999	21.98180205	77.69655262	**0**
Hepatitis B	1766	delayed exponetial	30.1266356	39.237536	22.92714454	70.37958515	**0**
Cranial nerve disease	1968	delayed exponetial	26.0631661	54.0574824	23.08953407	90.9490087	**0**
Mitochondrial complex I deficiency	438	delayed exponetial	50.0984136	29.3874018	23.196995	81.54264119	**0**
Charcot–Marie–Tooth disease type 4J	416	delayed exponetial	20.8782413	45.6880886	23.27107781	86.1850764	**0**
Pain agnosia	1866	delayed exponetial	43.9261611	48.4721003	23.34782215	87.36879237	**0**
Porphyria	536	delayed exponetial	36.0440932	50.009813	23.38743359	99.82220054	**0**
Isolated growth hormone deficiency	854	delayed exponetial	37.6919028	50.7127444	23.4246558	94.28303422	**0**
Stomach cancer	1613	delayed exponetial	30.4086342	39.0560754	23.45992392	68.99364424	**0**
Juvenile absence epilepsy	218	delayed exponetial	33.2824152	34.9695738	23.54231894	84.47316059	**0**
Glycogen storage disease IX	120	delayed exponetial	19.1630929	42.3124487	23.66654833	86.73121461	**0**

PPI network	Nodes	Best model	AIC_{min}	ΔAIC_f	ΔAIC_e	ΔAIC_{df}	ΔAIC_{de}
Blood coagulation disease	1959	delayed exponetial	25.8655329	44.2163823	23.75411084	75.8640642	**0**
Blood platelet disease	1954	delayed exponetial	31.744388	48.3556337	23.88150391	85.18786296	**0**
Primary bacterial infectious disease	1964	delayed exponetial	39.5379689	43.1250965	23.97872312	77.49572816	**0**
Tooth agenesis	704	delayed exponetial	44.8997536	40.1477507	24.00154084	83.51739047	**0**
Motor neuritis	152	delayed exponetial	15.5508659	36.12249	24.01598941	75.20934927	**0**
Renal coloboma syndrome	264	delayed exponetial	25.4378702	44.3819448	24.07364512	85.30947939	**0**
Hypertension	1919	delayed exponetial	32.7346302	47.9742821	24.3495753	83.94623332	**0**
Disease of metabolism	1964	delayed exponetial	24.6296134	45.0909797	24.41664574	77.13761594	**0**
Multiple carboxylase deficiency	294	delayed exponetial	27.6154046	40.7023836	24.5247423	85.03429934	**0**
Isolated growth hormone deficiency type IB	111	delayed exponetial	33.1163023	14.8100012	24.74396457	5.419411224	**0**
Multiple acyl-CoA dehydrogenase deficiency	376	delayed exponetial	29.7577988	32.3517594	25.07557485	75.56468471	**0**
Amelogenesis imperfecta type 1E	172	delayed exponetial	13.7532279	38.537956	25.26293044	79.0399659	**0**
Holocarboxylase synthetase deficiency	103	delayed exponetial	17.7754147	30.3590202	25.28125413	6.034589649	**0**
Pancreas disease	1827	delayed exponetial	36.7924267	43.2113714	25.62683302	79.23380883	**0**
Optic nerve disease	1961	delayed exponetial	23.6966437	56.6471106	25.9916289	93.269853	**0**
Autosomal dominant Alport syndrome	99	delayed exponetial	19.3861092	24.8180304	26.04197937	3.152641427	**0**
Tetanus	1695	delayed exponetial	30.2513306	50.2268371	26.40997363	84.67750401	**0**
Sjogren–Larsson syndrome	163	delayed exponetial	25.974461	30.9904442	26.53585574	75.87504973	**0**
Drug-induced lupus erythematosus	107	delayed exponetial	19.201158	28.6514469	26.62378099	4.575625301	**0**
Liver disease	1803	delayed exponetial	28.1402554	49.9893667	26.72205385	87.67417709	**0**
Colon cancer	1806	delayed exponetial	22.0574	37.4516799	27.0428847	63.51734828	**0**
Autosomal dominant cutis laxa	172	delayed exponetial	10.8406812	43.6423189	27.18116096	81.98677358	**0**
Charcot–Marie–Tooth disease type 4	949	delayed exponetial	23.8352073	60.9708189	27.30673287	96.65058892	**0**
Obesity	1931	delayed exponetial	26.8594019	43.9017488	27.42745961	74.71659748	**0**
Kidney failure	1930	delayed exponetial	29.3902225	50.9124527	27.46820845	87.37494192	**0**
Glycogen storage disease VII	184	delayed exponetial	14.2872165	38.055919	27.69695454	79.56938904	**0**
Alveolar echinococcosis	217	delayed exponetial	16.6044515	37.6194361	27.72004779	79.90053896	**0**
Connective tissue disease	1967	delayed exponetial	30.9720846	51.957081	28.13684764	86.03960517	**0**
Neuronal ceroid lipofuscinosis 8 northern epilepsy variant	133	delayed exponetial	25.7735431	38.6739616	28.57722599	81.99401237	**0**
Polycystic liver disease	439	delayed exponetial	7.88780747	52.5032802	28.78165937	87.91082957	**0**
Borjeson–Forssman–Lehmann syndrome	238	delayed exponetial	25.6855637	40.0442003	29.07229989	83.07009762	**0**
Cardiomyopathy	1861	delayed exponetial	19.0696615	49.8169659	29.08591136	82.04497173	**0**
Amyotrophic lateral sclerosis type 4	542	delayed exponetial	19.4523042	50.8549995	29.3959934	91.86969039	**0**
Hepatitis C	1750	delayed exponetial	22.2385583	47.2012657	29.4241674	78.14108701	**0**

PPI network	Nodes	Best model	AIC_{min}	ΔAIC_f	ΔAIC_e	ΔAIC_{df}	ΔAIC_{de}
Apoptosis	1017	delayed exponetial	53.8304333	35.0295999	29.51362391	46.37931305	**0**
Chronic progressive external ophthalmoplegia	800	delayed exponetial	15.9586875	61.3937611	29.63112674	101.6591277	**0**
Frontotemporal dementia	1891	delayed exponetial	28.3862954	54.0908621	29.85367916	88.07408885	**0**
Hematologic cancer	1873	delayed exponetial	28.486404	51.4628778	29.92738131	87.86943326	**0**
Cancer	1917	delayed exponetial	35.1167002	48.8999573	30.09392652	81.5709812	**0**
Colonic disease	1818	delayed exponetial	25.7590766	43.9073222	30.24218391	75.13802585	**0**
Viral hepatitis	1784	delayed exponetial	26.6672828	52.0675425	30.32132814	89.00569734	**0**
Nephronophthisis 1	187	delayed exponetial	61.2368761	34.8285438	30.40622142	103.604202	**0**
Progressive osseous heteroplasia	161	delayed exponetial	12.9107972	45.4498197	31.76146196	88.84107618	**0**
Hepatitis C2	1750	delayed exponetial	19.5485347	49.819343	31.88925042	80.83223252	**0**
Lupus erythematosus	1903	delayed exponetial	27.3506794	54.7367832	31.93561141	89.20535555	**0**
Pneumonia	1956	delayed exponetial	30.5201808	52.1163685	32.48301547	86.45463458	**0**
Anemia	1982	delayed exponetial	13.3034675	54.8533563	32.95282563	88.59355931	**0**
Brachydactyly type D	226	delayed exponetial	15.6964366	59.802876	32.97775246	103.0033289	**0**
Autosomal dominant disease	1968	delayed exponetial	12.1577449	57.8817168	33.17985874	89.59317169	**0**
Autosomal dominant nocturnal frontal lobe epilepsy	327	delayed exponetial	24.0148887	44.9670759	33.5544557	90.52701878	**0**
Hepatitis	1842	delayed exponetial	13.4175012	45.6790969	33.70320425	72.33888478	**0**
Fatty liver disease	1865	delayed exponetial	10.070716	59.2080586	33.72635839	91.03240376	**0**
Acute hemorrhagic conjunctivitis	161	delayed exponetial	19.4152874	44.3999832	33.77042747	92.24874696	**0**
Buschke–Ollendorff syndrome	170	delayed exponetial	8.82088792	40.9591051	34.03896646	83.7276407	**0**
Lipid metabolism disorder	1925	delayed exponetial	23.4178005	57.186962	34.78907988	93.31996739	**0**
Primary immunodeficiency disease	1974	delayed exponetial	22.2668901	60.4488135	35.45041462	94.79025276	**0**
Carbohydrate metabolic disorder	1931	delayed exponetial	17.8008626	52.2164849	36.4271473	83.80346646	**0**
Multiple sclerosis	1917	delayed exponetial	16.2461333	54.6713995	38.71268055	85.25332387	**0**
Llipid metabolism disorder	1925	delayed exponetial	18.8401428	61.687997	39.58298214	97.89883885	**0**
Trichohepatoenteric syndrome	243	delayed exponetial	15.6772266	47.9156996	39.90855352	93.41682092	**0**
Lleukemia	1857	delayed exponetial	14.2213915	55.702505	41.13058876	86.92119863	**0**
Glucose metabolism disease	1926	delayed exponetial	14.0680684	56.3702756	41.40847973	87.50832953	**0**
Alzheimer's disease	1932	delayed exponetial	21.4129045	59.8958816	41.49990759	95.40067531	**0**
Legg–Calve–Perthes disease	195	delayed exponetial	32.7546096	41.2572246	42.41565903	102.9734605	**0**
Degenerative myopia	214	delayed exponetial	16.0301143	53.0811181	43.35312627	101.3269418	**0**
Lysosomal storage disease	1926	delayed exponetial	6.7780167	63.6701432	44.02544883	94.74050866	**0**
Peritoneum cancer	409	delayed fractal	38.9385156	40.0221663	6.328862161	**0**	**0.52008509**
Schwartz–Jampel syndrome 1	93	delayed fractal	28.4687204	29.3829883	24.52435979	**0**	**0.78423076**

PPI network	Nodes	Best model	AIC_{min}	ΔAIC_f	ΔAIC_e	ΔAIC_{df}	ΔAIC_{de}
Third-degree atrioventricular block	230	delayed fractal	36.9074865	40.9221535	10.12287489	**0**	**1.490539345**
X-linked intellectual disability-psychosis-macroorchidism syndrome	107	delayed fractal	24.8615795	33.5461124	4.570408364	**0**	2.415619621
Brain ischemic RI	99	delayed fractal	6.87514967	12.4609416	**1.289960449**	**0**	2.44026215
Ischiocoxopodopatellar syndrome	97	delayed fractal	31.3727763	27.8373254	16.14157362	**0**	3.262022934
Multiple congenital anomalies-hypotonia-seizures syndrome	84	delayed fractal	31.724889	6.69023934	21.53385025	**0**	3.321537011
Heart aneurysm	203	delayed fractal	23.7445582	32.7091272	12.02005259	**0**	3.576688912
Myosin	100	delayed fractal	11.7236209	14.0853967	2.206482961	**0**	4.124058935
Immunodeficiency 43	84	delayed fractal	26.0799465	12.6314163	5.430773124	**0**	4.680919359
Immunodeficiency 23	151	delayed fractal	36.7359949	2.62611625	12.23670126	**0**	4.861451128
Hepatitis E	425	delayed fractal	40.8842265	45.9714267	16.29170013	**0**	5.193152173
Colon Cancer RI	95	delayed fractal	4.78353306	8.12933202	4.435207154	**0**	5.338593471
Brachydactyly type C	92	delayed fractal	20.7909829	29.7087145	31.21474066	**0**	5.507072144
Multiple chemical sensitivity	119	delayed fractal	29.8589614	18.5261427	5.520599915	**0**	5.606886311
EPICATECHIN 2	96	delayed fractal	1.40313088	20.9365862	9.116294958	**0**	5.613210202
Stroke	98	delayed fractal	15.7691871	13.7831216	4.521285887	**0**	5.667112442
Charcot–Marie–Tooth disease type 2A1	116	delayed fractal	22.5294738	23.6918893	3.949836308	**0**	5.918660338
Charcot–Marie–Tooth disease type 2I	114	delayed fractal	23.5200326	26.4112451	21.8919573	**0**	5.967041045
Osteogenesis imperfecta type 15	104	delayed fractal	21.5514495	32.1892205	25.57352436	**0**	5.973153739
IgG4-related disease	256	delayed fractal	22.7737543	39.3906539	5.561915346	**0**	6.123425047
Intermittent explosive disorder	113	delayed fractal	18.8639545	37.7425631	13.04690909	**0**	6.322743535
SAPHO syndrome	163	delayed fractal	15.6498955	29.0126398	6.831730025	**0**	6.565810876
Immunodeficiency 16	162	delayed fractal	22.8029075	16.9247327	7.782051007	**0**	6.659742715
Syndactyly type 4	102	delayed fractal	22.4262703	16.0274928	8.235441791	**0**	7.007164679
Myocardial infarction RI	99	delayed fractal	6.52437698	20.5564583	5.294515994	**0**	7.054272346
Autosomal recessive osteopetrosis 2	136	delayed fractal	29.2856077	25.7004778	6.327012504	**0**	7.167213731
Multiple symmetric lipomatosis	89	delayed fractal	18.0139111	22.7828564	10.93476504	**0**	7.437999782
Brachydactyly type B1	121	delayed fractal	15.3956314	23.1524447	5.920642673	**0**	7.725554264
Immunodeficiency with hyper IgM type 3	207	delayed fractal	14.3737944	32.7074656	6.061089732	**0**	7.786892831
Small intestine neuroendocrine neoplasm	123	delayed fractal	27.9857283	29.8578053	6.486490831	**0**	7.908662002
Charcot–Marie–Tooth disease type 1D	160	delayed fractal	27.5818983	32.0891797	6.190790922	**0**	8.001574003
COVID-19	85	delayed fractal	4.48083532	21.7441854	6.171046247	**0**	8.079161059
Immunodeficiency 73a with defective neutrophil chemotaxis and leukocytosis	103	delayed fractal	31.9586779	25.8873951	9.243369986	**0**	8.222460915
Cervix small cell carcinoma	38	delayed fractal	5.23378581	22.8966949	14.17199288	**0**	8.234020244
Mikulicz disease	113	delayed fractal	8.87868877	26.9127919	11.01205528	**0**	8.360213113

PPI network	Nodes	Best model	AIC_{min}	ΔAIC_f	ΔAIC_e	ΔAIC_{df}	ΔAIC_{de}
Arterial calcification of infancy	171	delayed fractal	32.3895763	30.6353088	10.59587497	**0**	8.430525865
Scarlet fever	76	delayed fractal	23.5264503	19.0044838	15.54091341	**0**	8.464610248
Familial medullary thyroid carcinoma	154	delayed fractal	22.0547306	30.7265309	11.40818596	**0**	8.87400317
Autosomal dominant Emery-Dreifuss muscular dystrophy 4	123	delayed fractal	24.7507593	27.740993	7.725637679	**0**	8.897763699
Charcot–Marie–Tooth disease dominant intermediate C	186	delayed fractal	35.1694307	28.0759348	9.933053941	**0**	8.921524332
Immunodeficiency 35	224	delayed fractal	14.6424409	22.8407522	13.95936945	**0**	9.290073538
Charcot–Marie–Tooth disease type 2A2A	99	delayed fractal	29.7879107	22.4185201	9.298388349	**0**	9.819169412
Factor XI deficiency	168	delayed fractal	18.3612386	28.8147361	12.93803089	**0**	10.23166599
Autosomal dominant auditory neuropathy 1	168	delayed fractal	28.4414071	31.1853682	9.042359497	**0**	10.50670375
Egg allergy	192	delayed fractal	29.6022657	25.478025	9.206132396	**0**	10.60552579
Immunodeficiency 50	86	delayed fractal	15.6080217	23.2606948	37.04570787	**0**	10.74137752
Intermittent asthma	150	delayed fractal	34.4254275	27.4362263	16.43547938	**0**	11.14525117
Immunodeficiency 40	219	delayed fractal	28.1950483	29.7038594	9.8864428	**0**	11.32358219
Hereditary spastic paraplegia 18	129	delayed fractal	18.5777817	30.5937711	17.20439655	**0**	11.34844844
Autosomal dominant hypocalcemia	200	delayed fractal	38.2817654	30.9010804	11.41369267	**0**	11.44262392
Amelogenesis imperfecta type 3	124	delayed fractal	28.1650477	34.0119888	20.97534879	**0**	11.96692125
Osteogenesis imperfecta type 12	92	delayed fractal	20.609847	28.6109445	32.67367995	**0**	12.46992485
Charcot–Marie–Tooth disease axonal type 2CC	88	delayed fractal	15.3292454	30.0900546	33.37957291	**0**	12.85596754
Charcot–Marie–Tooth disease dominant intermediate E	106	delayed fractal	12.2719479	34.6363538	16.71819028	**0**	13.11657742
Interstitial keratitis	96	delayed fractal	12.1336992	32.5714364	20.03656114	**0**	13.33857257
Multiple pterygium syndrome	128	delayed fractal	27.5228403	35.435181	24.91467475	**0**	13.40884401
Osteogenesis imperfecta type 2	80	delayed fractal	27.2505521	21.631421	24.33412183	**0**	13.51356871
Drug allergy	281	delayed fractal	16.6732483	37.6178387	11.55125615	**0**	13.51677324
Charcot–Marie–Tooth disease axonal type 2N	161	delayed fractal	20.5271779	34.6467554	12.56157862	**0**	13.52520482
Progressive relapsing multiple sclerosis	68	delayed fractal	6.44631636	22.8343188	28.34094279	**0**	13.58137194
Intermediate spinal muscular atrophy	170	delayed fractal	16.1081674	41.8889002	11.90746618	**0**	13.84856651
Primary autosomal recessive microcephaly 2 with or without cortical malformations	146	delayed fractal	20.6190761	28.6369426	27.17435374	**0**	13.89868867
Brachydactyly type A2	169	delayed fractal	17.8973271	40.4723495	16.55086982	**0**	14.20842933
Duodenum adenocarcinoma	176	delayed fractal	21.2216061	44.3157366	25.26734103	**0**	14.49891739
Pericarditis	599	delayed fractal	32.0621121	44.3744222	14.57104448	**0**	14.52267698
Diabetes Mellitus RI	90	delayed fractal	-4.4774567	32.9370241	15.29703302	**0**	14.75374636
Immunodeficiency 11A	135	delayed fractal	26.364837	29.9233535	21.43014955	**0**	14.76140668
Osteochondritis dissecans	268	delayed fractal	27.6622734	39.1722811	17.8325412	**0**	14.96908189
Primary failure of tooth eruption	85	delayed fractal	12.9090915	24.8688167	37.44905904	**0**	15.78359179

PPI network	Nodes	Best model	AIC_{min}	ΔAIC_f	ΔAIC_e	ΔAIC_{df}	ΔAIC_{de}
Syndactyly type 3	157	delayed fractal	26.2020767	29.5410505	23.55614397	**0**	16.70571356
Left bundle branch hemiblock	294	delayed fractal	35.9471498	39.3293818	15.06503876	**0**	16.92768445
Autosomal dominant microcephaly	99	delayed fractal	38.9572951	6.46845465	41.70795807	**0**	17.11376782
Colon mucinous adenocarcinoma	94	delayed fractal	5.61091377	38.204731	15.47320805	**0**	17.46187771
Autosomal dominant nonsyndromic deafness 10	141	delayed fractal	11.9238024	40.9718287	18.73798188	**0**	17.57866971
CINCA Syndrome	414	delayed fractal	29.5149435	44.4726251	16.70629873	**0**	17.70683242
Amelogenesis imperfecta type 1B	125	delayed fractal	9.98476936	35.2139709	22.76855552	**0**	17.79143019
Nasu–Hakola disease	378	delayed fractal	27.9313422	42.5427735	21.0135111	**0**	18.37782006
Small intestine benign neoplasm	167	delayed fractal	10.3033628	44.4069456	26.19723754	**0**	18.78277063
Thyroid hormone resistance syndrome	171	delayed fractal	23.1496064	40.5930464	16.84125632	**0**	18.79691058
Syndromic microphthalmia 9	174	delayed fractal	18.984599	52.7278509	21.3360676	**0**	19.75705588
Multiple benign circumferential skin creases on limbs	86	delayed fractal	2.38729057	37.9842513	29.42346193	**0**	19.76241394
Viral exanthem	169	delayed fractal	15.9499202	40.0508972	27.1075461	**0**	20.60473475
Osteoglophonic dysplasia	163	delayed fractal	15.5481498	46.893675	19.39112741	**0**	20.61894082
Pool Akt AMPK mTORparamat	34	delayed fractal	-9.4407705	4.33315464	20.56023323	**0**	21.20889279
Brachydactyly-syndactyly syndrome	93	delayed fractal	12.9744716	21.0164239	32.18791963	**0**	21.71449891
Cenani–Lenz syndactyly syndrome	121	delayed fractal	16.6402441	47.184613	36.49484733	**0**	21.89887607
Drug-induced hepatitis	185	delayed fractal	2.93957867	48.4051532	21.69520151	**0**	22.5286208
Blood group incompatibility	115	delayed fractal	20.2854872	43.8383463	25.98513184	**0**	22.55485158
Gnathodiaphyseal dysplasia	189	delayed fractal	22.119587	50.967671	24.19693934	**0**	23.22524021
Charcot–Marie–Tooth disease type 4E	134	delayed fractal	8.85255565	51.4635137	23.5471242	**0**	23.57199284
Hepatitis D	248	delayed fractal	25.7698808	52.2634605	24.13794667	**0**	26.07302066
Peutz–Jeghers syndrome	610	delayed fractal	28.9293949	62.0668983	27.62062849	**0**	26.48262379
Charcot–Marie–Tooth disease type 4K	128	delayed fractal	-11.037854	43.8288608	31.31798005	**0**	26.63200375
Apparent mineralocorticoid excess syndrome	113	delayed fractal	2.16802552	48.8585166	33.89355978	**0**	26.75964744
Neonatal abstinence syndrome	113	delayed fractal	26.0914439	26.0041484	54.35468941	**0**	27.71876986
Collecting duct carcinoma	272	delayed fractal	13.8263639	60.8685683	29.00704509	**0**	27.9399737
Cervix disease	1639	delayed fractal	18.8273053	69.2010662	45.42888152	**0**	30.43074924
Thyroid gland anaplastic carcinoma	625	delayed fractal	27.6232293	66.0469044	33.4674742	**0**	32.35699355
Bruck syndrome	204	delayed fractal	13.208252	63.633288	36.83995821	**0**	32.49887626
Tibial muscular dystrophy	336	delayed fractal	17.6959786	70.509316	34.64115419	**0**	36.5499124
Restrictive cardiomyopathy	432	exponential	43.7597739	30.4663597	**0**	75.93643928	**0.045622647**
Charcot–Marie–Tooth disease type 4B3	256	exponential	37.644796	25.7585048	**0**	61.6748221	**0.062410188**
Iinterstitial nephritis	575	exponential	38.7641291	31.3846857	**0**	73.55653983	**0.180500127**

PPI network	Nodes	Best model	AIC_{min}	ΔAIC_f	ΔAIC_e	ΔAIC_{df}	ΔAIC_{de}
Thyroid gland adenocarcinoma	756	exponential	43.4514498	45.9690386	**0**	86.46100435	**0.22239321**
Thyroid adenoma	795	exponential	53.1248653	37.72494	**0**	77.72446223	**0.291881408**
Adult acute lymphocytic leukemia	889	exponential	51.1454424	35.9972221	**0**	81.5634768	**0.345109089**
Charcot–Marie–Tooth disease type 2D	314	exponential	34.5680827	32.6004858	**0**	68.05840106	**0.40470343**
Colorectal adenoma	1485	exponential	63.4159026	34.2294481	**0**	78.54597689	**0.446311428**
Autosomal dominant nonsyndromic deafness 1	214	exponential	22.6612472	39.4266664	**0**	73.88889497	**0.472776719**
Charcot–Marie–Tooth disease type 2B2	164	exponential	20.5704124	29.3168388	**0**	61.41474022	**0.536593932**
Choroideremia	440	exponential	43.155704	37.1774511	**0**	76.96176996	**0.538030052**
Thyroid gland cancer	1281	exponential	48.3766122	37.9976706	**0**	76.83825597	**0.565572322**
Recessive dystrophic epidermolysis bullosa	383	exponential	31.1252225	37.2923041	**0**	74.71361447	**0.605641886**
Hair disease	1952	exponential	50.091499	40.6682612	**0**	81.83746326	**0.826309477**
Ollier disease	249	exponential	35.382923	37.81925	**0**	74.58743889	**0.849348932**
Endogenous depression	1762	exponential	58.6771065	46.690308	**0**	86.50001582	**0.861608021**
Autosomal dominant polycystic kidney disease	1417	exponential	47.0629687	39.985571	**0**	79.74452999	**0.870663982**
Smallpox	727	exponential	45.0138337	30.7779329	**0**	71.11183219	**0.932016948**
Autosomal dominant nonsyndromic deafness	617	exponential	50.77889	36.2776893	**0**	75.45918645	**0.957918969**
Charcot–Marie–Tooth disease type 3	219	exponential	41.0972926	29.3528802	**0**	66.46102558	**1.01841573**
Charcot–Marie–Tooth disease type 4A	264	exponential	20.6055918	43.516643	**0**	79.28003251	**1.191120811**
Ovary adenocarcinoma	1609	exponential	77.0175202	51.7428322	**0**	109.6220568	**1.191576459**
Thyroid dyshormonogenesis	330	exponential	35.6318242	26.0828777	**0**	56.21162362	**1.277088047**
Thyroid gland carcinoma	1238	exponential	48.2431636	36.4529043	**0**	76.38759931	**1.280690964**
Adrenal adenoma	541	exponential	33.8261604	39.491159	**0**	77.55177802	**1.287774188**
Multiple endocrine neoplasia	796	exponential	28.2111669	48.3670852	**0**	89.35947525	**1.32134277**
Intrahepatic cholestasis	759	exponential	59.6711189	37.2616951	**0**	83.28605885	**1.328738851**
Charcot–Marie–Tooth disease axonal type 2P	196	exponential	22.5330392	31.6024585	**0**	62.00584176	**1.336825107**
Brugada syndrome	522	exponential	52.8709844	27.2369336	**0**	70.26677303	**1.336932632**
Progressive familial intrahepatic cholestasis	357	exponential	47.3783439	20.535543	**0**	57.24855994	**1.392975043**
Progressive muscular atrophy	312	exponential	40.6615842	29.1931133	**0**	61.95284947	**1.401657265**
Colon adenoma	768	exponential	71.2171121	40.2083537	**0**	46.5467251	**1.47666175**
Transthyretin amyloidosis	515	exponential	35.3660793	36.4839231	**0**	75.21847606	**1.557032006**
Charcot–Marie–Tooth disease type 1	85	exponential	10.8896603	18.9038533	**0**	7.831311177	**1.589263013**
Ventricular septal defect	1657	exponential	60.2543793	39.4119263	**0**	83.69719527	**1.633209389**
Charcot–Marie–Tooth disease type 1C	238	exponential	34.8369111	28.9613721	**0**	63.39966509	**1.646291129**
Fibrodysplasia ossificans progressiva	361	exponential	26.8059179	29.8240655	**0**	66.3431684	**1.716881578**

PPI network	Nodes	Best model	AIC_{min}	ΔAIC_f	ΔAIC_e	ΔAIC_{df}	ΔAIC_{de}
Blood protein disease	1443	exponential	46.1827023	40.8786604	**0**	80.90547129	**1.744756226**
Autosomal dominant hypophosphatemic rickets	204	exponential	24.2473316	29.0070682	**0**	60.85679474	**1.755474002**
Endocarditis	1004	exponential	41.8111889	41.4911177	**0**	79.47990566	**1.77779686**
Cowden syndrome	586	exponential	44.1776499	27.4197611	**0**	68.44915925	**1.8234556**
Carotid artery disease	748	exponential	40.3632897	35.9668795	**0**	76.20656928	**1.830095062**
Charcot–Marie–Tooth disease type 1B	268	exponential	30.9792702	33.7369425	**0**	69.13380591	**1.840862704**
Autosomal dominant limb-girdle muscular dystrophy type 1	223	exponential	23.3552488	29.7015172	**0**	63.00205279	**1.844967368**
Hepatocellular adenoma	477	exponential	32.2415879	30.1122178	**0**	64.79928806	**1.8533748**
Vici syndrome	340	exponential	24.3921174	37.3137034	**0**	67.84270043	**1.88307607**
Charcot–Marie–Tooth disease dominant intermediate B	253	exponential	17.3809901	39.318053	**0**	70.76061373	**1.885356811**
Pneumoconiosis	746	exponential	34.7854967	31.5538713	**0**	68.5574043	**1.887958005**
Iron deficiency anemia	590	exponential	32.2924949	33.6990945	**0**	67.72275175	**1.905781815**
Kidney angiomyolipoma	244	exponential	18.494578	29.3313756	**0**	58.00055808	**1.911377925**
Huntington's disease like	86	exponential	5.842199	30.3706774	**0**	2.576049137	**1.914434935**
Syndactyly	1654	exponential	57.6812574	53.6180636	**0**	100.840398	**1.932239008**
Moyamoya disease	396	exponential	56.3662707	32.5355937	**0**	73.72921644	**1.936470723**
Autosomal dominant limb-girdle muscular dystrophy	277	exponential	24.8426301	24.7833189	**0**	53.1342206	**1.975299881**
Achondroplasia	634	exponential	47.4541997	37.926237	**0**	79.25703103	**1.977080868**
Renal hypoplasia	477	exponential	29.3373513	44.5677381	**0**	80.03680555	**1.981115615**
Multiple intestinal atresia	145	exponential	21.4521899	30.0217901	**0**	58.81148396	**1.98120947**
Lipoma	686	exponential	51.4868846	34.9331582	**0**	76.62885912	**1.981402169**
X-linked agammaglobulinemia	607	exponential	32.3910672	31.8610449	**0**	68.06193933	**1.981503988**
Primary biliary cholangitis	1494	exponential	41.6826147	45.0703198	**0**	85.96010746	**1.988532016**
Syndromic microphthalmia	453	exponential	66.3547092	32.5068546	**0**	78.3467242	**1.990510708**
Osteoblastoma	273	exponential	31.6081782	25.3143641	**0**	57.57057935	**1.992343725**
Thyroid gland follicular carcinoma	753	exponential	40.2550214	48.614155	**0**	89.56523698	**1.993817926**
Small intestine cancer	475	exponential	29.6720493	34.1389939	**0**	67.30200466	**1.997500404**
Motor peripheral neuropathy	607	exponential	35.7892175	51.0279024	**0**	90.19259275	**1.999589044**
Viral infectious disease	1927	exponential	55.7550318	15.1712407	**0**	45.81521421	7.99952388
Lymphatic system disease	1855	exponential	54.6717839	15.6936489	**0**	46.42791289	8.348961071
Schizophrenia	1919	exponential	55.0960845	17.8169366	**0**	46.34574641	10.98916521
Pulmonary fibrosis	1831	exponential	57.9686942	22.4065073	**0**	58.05670702	12.25680437
Juvenile myelomonocytic leukemia	660	exponential	40.2465947	20.1848325	**0**	48.3325473	13.26543118
Small cell carcinoma	834	Exponential	49.254881	22.8454376	**0**	55.75074129	14.1210068

**PPI network**	**Nodes**	**Best model**	**AIC_{min}**	**ΔAIC_f**	**ΔAIC_e**	**ΔAIC_{df}**	**ΔAIC_{de}**
Multiple system atrophy	823	Exponential	46.1882369	25.9339226	**0**	58.57705967	16.94251258
Renal hypertension	572	Exponential	31.2953017	32.9370962	**0**	68.34673649	20.91742636
Alcohol dependence	1874	Exponential	47.2786961	34.4125668	**0**	68.97586491	22.90445355
Mitochondrial myopathy	1397	Exponential	39.8799445	44.6806601	**0**	86.68565146	25.70112006
Glycogen metabolism disorder	1485	Exponential	39.4202122	47.8039335	**0**	88.15812295	32.6498856
Autophagy	10130	delayed exponetial	77.593567	55.7170079	52.9279988	63.5056516	**0**
Glioblastoma	30041	delayed exponetial	91.5119539	60.2804332	57.0259197	68.8178781	**0**

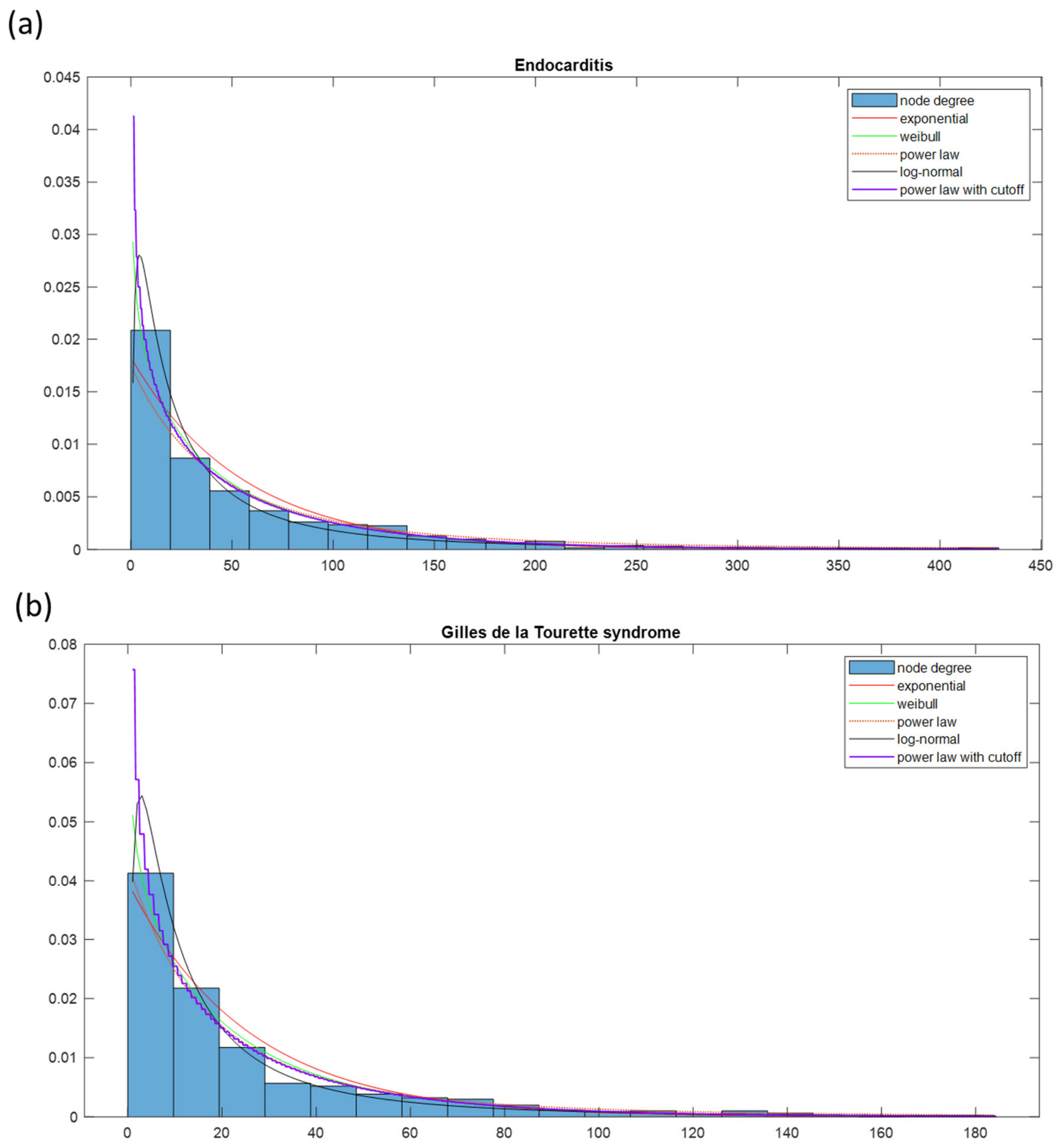

Figure S1. The fit of several models for the node degree probability distribution of (a) Endocarditis network and (b) Gilles de la Tourette syndrome network.

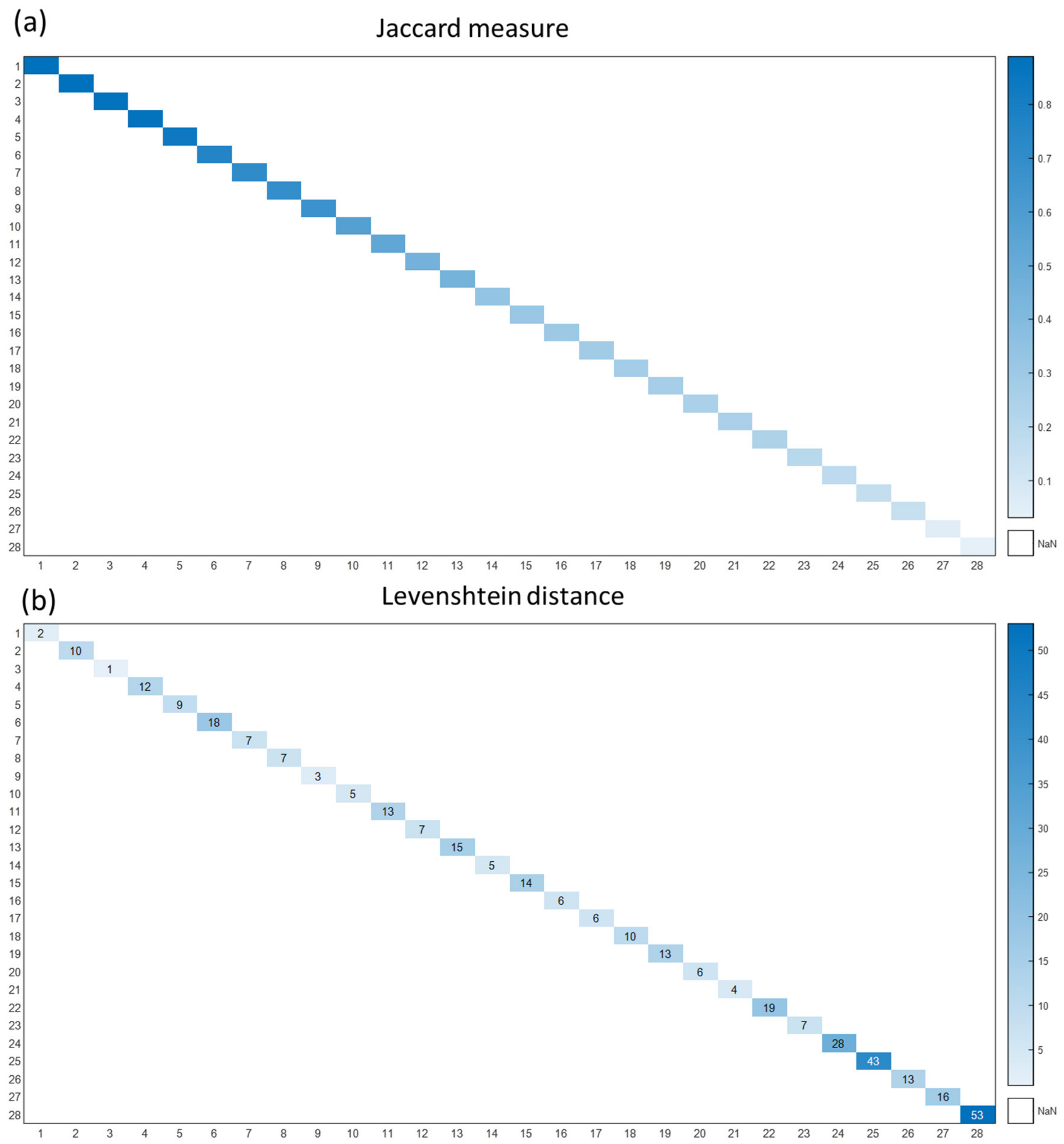

Figure S2. The (a) Jaccard measure and (b) Levenshtein distance between real and generated sequences of Immune

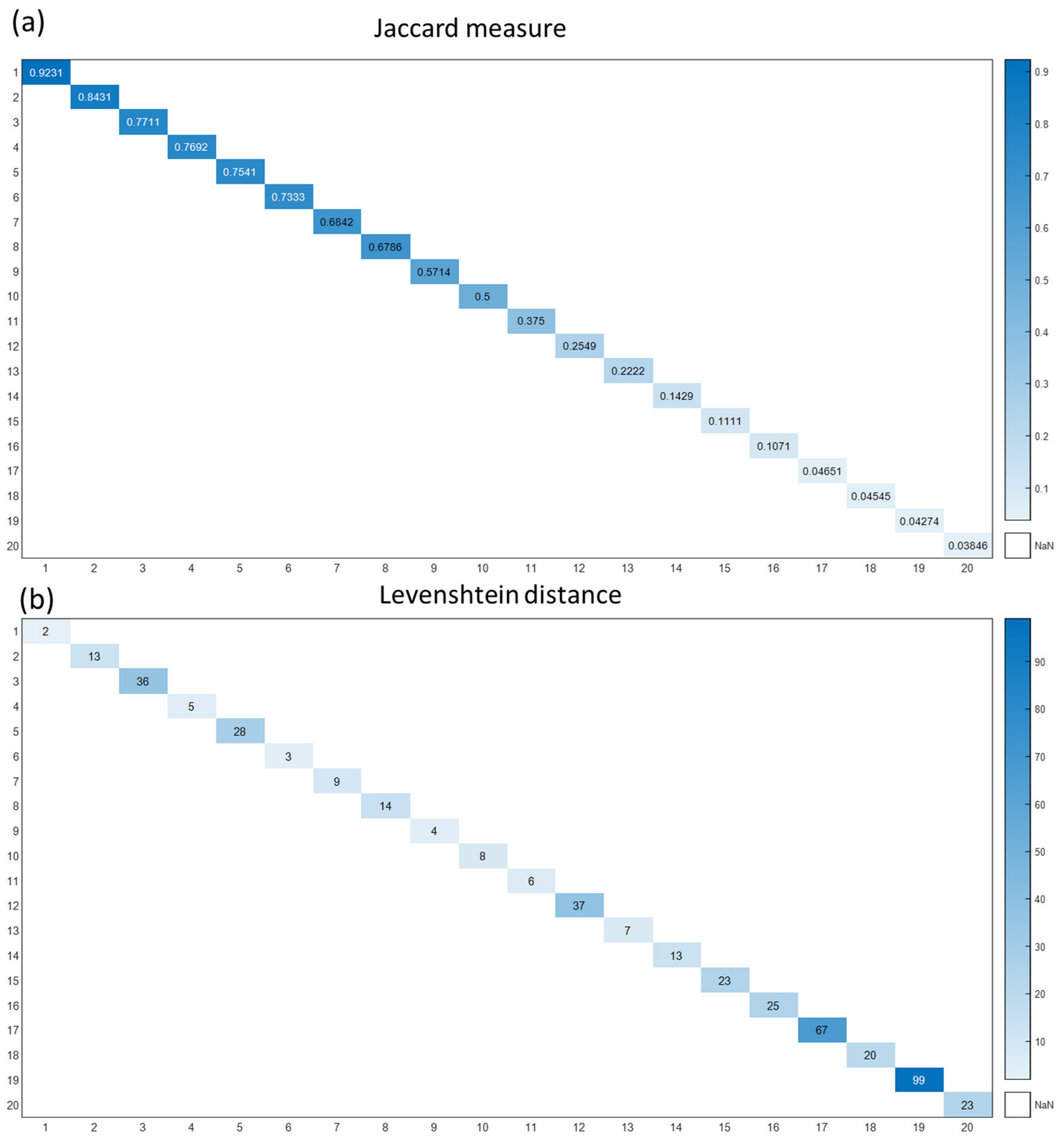

Figure S3. The (a) Jaccard measure and (b) Levenshtein distance between real and generated sequences of Metabolism

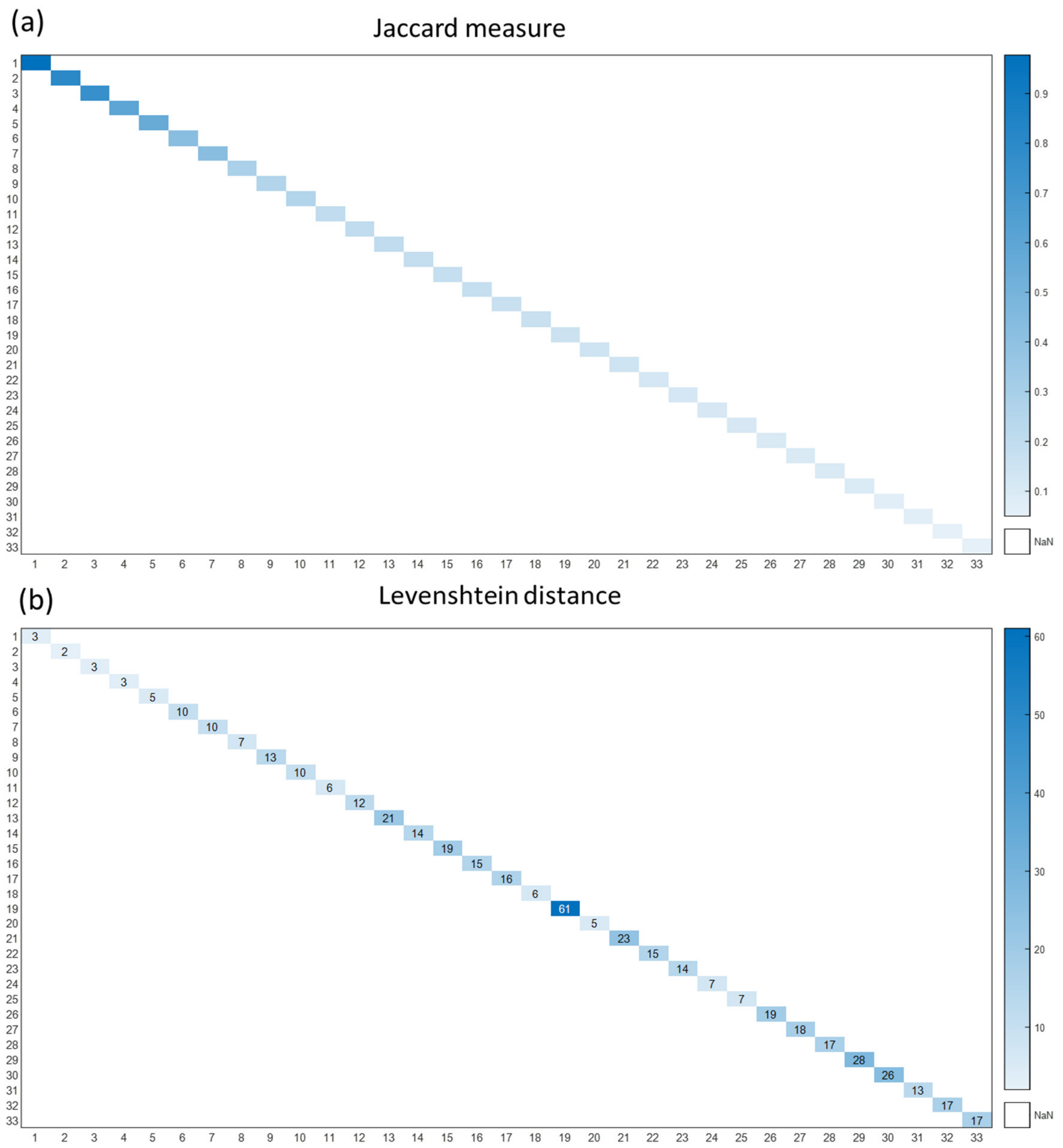

Figure S4. The (a) Jaccard measure and (b) Levenshtein distance between real and generated sequences of Motor

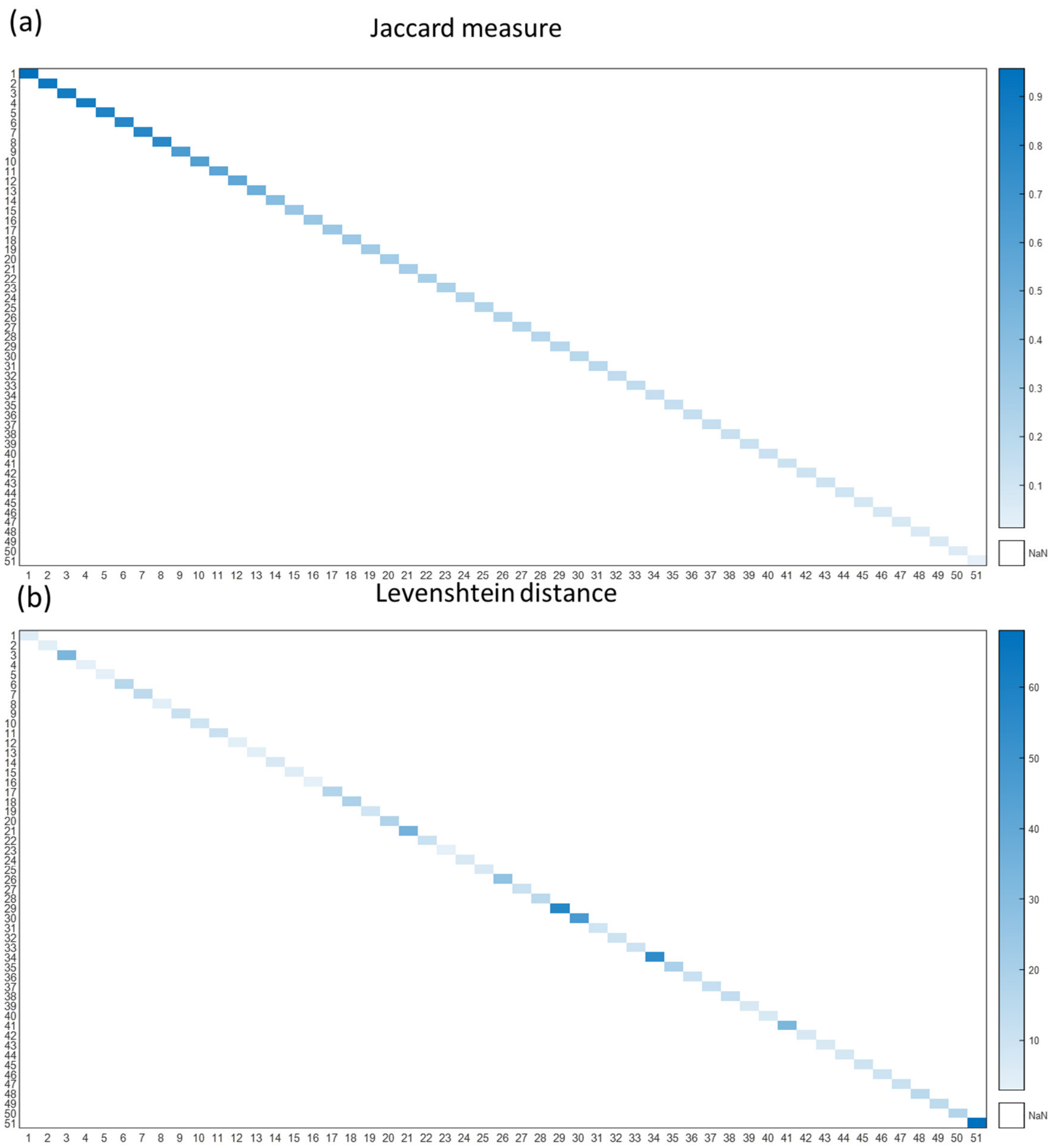

Figure S5. The (a) Jaccard measure and (b) Levenshtein distance between real and generated sequences of Nerve

Box-covering implementation

A brief example of the use of the box-covering code (shown below) computed on the network of Figure S6 (a) (represented by the adjacency matrix A) starts randomly selecting node three as the initial one (line 19 of the code). The column and row three are swapped with the column and row one (lines 20-41) of adjacency matrix A since the code takes the initial node as that in the first column and row. The NumberBoxes (line 42) function computes the minimum number of boxes to cover the network; the size of the boxes is between one to the diameter+1 of the network. In our example, the diameter is four. This function needs the number of nodes (nnodes), the diameter+1 of the networks and a squared matrix (that contains the shortest path distance from two nodes in the network).

(a)

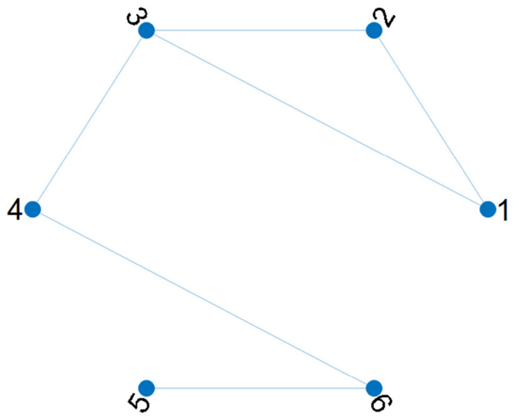

(b)

Node \ Box size	1	2	3	4	5
1	0	0	0	0	0
2	**1**	-1	-1	-1	-1
3	-1	-1	-1	-1	-1
4	-1	-1	-1	-1	-1
5	-1	-1	-1	-1	-1
6	-1	-1	-1	-1	-1

(c)

Node \ Box size	1	2	3	4	5
1	0	0	0	0	0
2	1	**0**	-1	-1	-1
3	-1	-1	-1	-1	-1
4	-1	-1	-1	-1	-1
5	-1	-1	-1	-1	-1
6	-1	-1	-1	-1	-1

(d)

Node \ Box size	1	2	3	4	5
1	0	0	0	0	0
2	1	0	0	0	0
3	2	0	0	0	0
4	3	1	0	0	0
5	4	-2	1	1	0
6	5	1	1	0	0

Figure S6. The box-covering implementation example of (a) brief network. (b) The result of the box number for node two for a size one. (c) The result of the box number for node two for a size two. (d) The box assignment for the network's six nodes for the box size from one to five.

This function computes the Cmin (n x m matrix, where n is the number of nodes and m =diameter+1) that summarises the box to that one node belongs given a box size. For example, column one of Cmin contains a set of integers in each entry representing the box (of size one) to which a given node(row) belongs. It is a trivial case where each node belongs to a different box (of size one); thus, column vector one will contain n different numbers representing the boxes; in our example, six boxes of size one cover the network. The computation of the Cmin by the code starts initializing the matrix with -1 (lines 65-67). The start node always is assigned to the box with the number zero for every box size; thus, row one of the Cmin matrix is set to zero (line 68). Next, a box for the rest of the nodes is assigned (loop of line 69) for every box size (loop of line 71). It is equivalent to filling the row vector with the corresponding box starting from node two. The unusedcolor function carries out this task. Following our example, the current node is the two (line 70), and the box size is one (lb=1, line 71), so the unusedcolor function obtains the distance to the other nodes numbered less than the current node (loop of line 90). If the distance is equal to or greater than the size of the box (line 94), the current node (two) must be assigned to a different box (using the next integer) (lines 95 and 96). In our code, the vector usedcolors stores the index of the boxes that have been used. In our example, the distance between nodes two and three (the node tree was swapped in the adjacency matrix with node one) is one, so node two must be assigned to the next box. Since box zero was assigned to node three (this value was marked in usedcolors vector), the next box is that numbered with one (obtained by lines 104-106). Next, this value is assigned in the Cmin(2,1)=1, as shown in Figure S6 (b).

Now, the box size is increased (lb=2, line 71); thus, the distance between nodes two and three fails the condition of line 94; thus, both nodes belong to the same box; in this case, box zero; hence Cmin(2,2)=0, see Figure S6 (C). The lb is increased again (lb=3, line 71) until lb=5; for these steps, the distance between node two and three is checked and fail the condition of line 94, so node 2 belongs to box zero for lb=[3,5]. The current node is moved to three (line 70 and lb=1 line 71) (numbered as 1 in Figure S6 (a) because of swapping). In this step of the loop of line 71, the unusedcolor function checks the distance between nodes 1,2,3 that are equal to one and equal to lb=1. Node three must belong to the next box available, the number two; see Figure S6 (d). The previous steps are repeated for the remaining nodes to produce the result in Figure S6 (d). The max value plus one of each column of Cmin is the minimum number of boxes of size equal to the number of the column (lines 49-50)

Code

In this section, the code of the box-covering network is listed. An example of the code to generate a new protein sequence can be downloaded from https://osf.io/pt7nj. Also, the example data for box-covering and protein sequence generation are included.

```
1   %Input%
2   % A: adjacency matrix of an undirected network
3   %Output %
4   %l: the diameter of the boxes [1, Diameter+1]
5   %Nb: number of the minimum boxes of diameter l to cover the network
6   %Cmin matrix that indicates the box of each node
7   % Please see [69] for in-deep details of the algorithm
8   function [l,Nb,Cmin]=boxcovering(A)
9    nnodes=size(A,1); %nnodes=number of nodes of the network
10   G=graph(A); %G=graph representation in MATLAB
11   d=distances(G,'Method','unweighted'); %d contains all the shortest paths from each node to other
12   diameter=max(d(isfinite(d)));  %diameter of the network
13   Aor=A;
14   dor=d;
15   A=Aor;
```

```
 d=dor;
 %we simulate that  the first node was picked out randomly
 % by changing the adjacency matrix row and column
 rvertex=randi(nnodes);
 d1=d(1);
 d2=d(rvertex);
 d(1)=d2;
 d(rvertex)=d1;
 for i=1:nnodes
     r1=A(1,i);
     rr=A(rvertex,i);
     A(1,i)=rr;
     A(rvertex,i)=r1;
 end
 for i=1:nnodes
     c1=A(i,1);
     cr=A(i,rvertex);
     A(i,1)=cr;
     A(i,rvertex)=c1;
 end
 firstc=d(:,1);
 d(:,1)=d(:,rvertex);
 d(:,rvertex)=firstc;
 firstr=d(1,:);
 d(1,:)=d(rvertex,:);
 d(rvertex,:)=firstr;
 Cmin=NumberBoxes(nnodes,diameter+1,d);  %Computes the minimun number of boxes (of diameter l) to cover the network.
 %change the vertex to the final position
 for w=1:length(Cmin(1,:))
     aux=Cmin(1,w);
     Cmin(1,w)=Cmin(rvertex,w);
     Cmin(rvertex,w)=aux;
 end
 Nb=max(Cmin);   %Obatains the number of boxes vector for each size l
 Nb=Nb+1;
 l=1:length(Nb); %Computed the size of the boxes  usually [1, diameter+1]
 l=transpose(l);
 Nb=transpose(Nb);
end
%computes the number of boxes from l=1 to lbmax
%Input%
```

```
%% nnodes: number of nodes in the network
%%lbmax: max diameter of the boxes, usually diameter +1
%d: minimum distance between nodes of the network
%Output%
%colors:Matrix of size= number of nodes X diamter+1.  Indicates the nodes (rows)
% colour (box) for a given distance l (column)
function Cmin= NumberBoxes(nnodes,lbmax,d)
%creates matrix #nodes*lbmax distance lbmax
Cmin=zeros(nnodes,lbmax); %note that -1 indicates no color
Cmin=Cmin-1;%this is to remark that value -1 means that a node have not been coloured yet
% colouring actual vertex with 0
Cmin(1,:)=0;
for i=2:nnodes
    actualvertex=i;
    for lb=1:lbmax
        ucolor=unusedcolor(actualvertex,lb,d,c);
        if ucolor~=-1 %uclor =-1  means that any distance lij was less than lb
            Cmin(actualvertex,lb)=ucolor;
        else
            Cmin(actualvertex,lb)=0;
        end
    end
end

end

%finds the unused color for a given lb
 function  color= unusedcolor( i, lb, distances,c)
 nnodes=size(c);
 nnodes=nnodes(1,1);
 color=-1;
 distancechecked=0;
 usedcolors=zeros(nnodes,1);
 for j=1:i-1
     if isinf(distances(i,j))
         continue;
     end
     if distances(i,j)>=lb
        ucolor=c(j,lb);
        usedcolors(ucolor+1,1)=1;
        distancechecked=distancechecked+1;
```

```
        end
    end
    %search for the first value of used colour set to 0
    if distancechecked==0 %any distance was >=lb so color must be 0
       color=0;
    else
        [i,~]=find(usedcolors~=1);
        if ~isempty(i)
            color=i(1)-1;
        end
    end
    end
```
